# Evidence on the performance of nature-based solutions interventions for coastal protection in biogenic, shallow ecosystems: a systematic map

**DOI:** 10.1186/s13750-024-00350-5

**Published:** 2024-12-02

**Authors:** Avery B. Paxton, Trevor N. Riley, Camille L. Steenrod, Brandon J. Puckett, Jahson B. Alemu I., Savannah T. Paliotti, Alyssa M. Adler, Laura Exar, Josette E. T. McLean, James Kelley, Y. Stacy Zhang, Carter S. Smith, Rachel K. Gittman, Brian R. Silliman

**Affiliations:** 1 National Centers for Coastal Ocean Science, National Ocean Service, National Oceanic and Atmospheric Administration, 101 Pivers Island Road, Beaufort, NC 28516 USA; 2 Central Library, Office of Science Support, Oceanic and Atmospheric Research, National Oceanic and Atmospheric Administration, 1315 East-West Highway, Silver Spring, MD 20910 USA; 3 CSS Inc., 10301 Democracy Lane, Suite 300, Fairfax, VA 22030 USA; 4 Stokes School of Marine and Environmental Sciences, University of South Alabama, Mobile, AL 36608 USA; 5 Department of Marine, Earth, and Atmospheric Sciences, North Carolina State University, 2800 Faucette Drive, Raleigh, NC 27695 USA; 6 Duke University Marine Lab, 135 Marine Lab Road, Beaufort, NC 28516 USA; 7 School of Aquatic and Fishery Sciences, University of Washington, 1122 NE Boat Street, Seattle, WA 98195 USA; 8 Department of Biology, East Carolina University, 101 E. 10th Street, Greenville, NC 27858 USA; 9 Coastal Studies Institute, East Carolina University, Wanchese, NC 850 NC 34527981 USA; 10 Present Address: Southeast Fisheries Science Center, National Marine Fisheries Service, National Oceanic and Atmospheric Administration, 101 Pivers Island Road, Beaufort, NC 28516 USA

**Keywords:** Artificial structure, Coastal hazard, Coastal resilience, Ecological engineering, Green infrastructure, Living shoreline, Natural and nature-based feature, Natural infrastructure, Nature-based infrastructure, Restoration

## Abstract

**Background:**

Combined impacts from anthropogenic pressures and climate change threaten coastal ecosystems and their capacity to protect communities from hazards. One approach towards improving coastal protection is to implement “nature-based solutions” (NBS), which are actions working with nature to benefit nature and humans. Despite recent increases in global implementation of NBS projects for coastal protection, substantial gaps exist in our understanding of NBS performance. To help fill this gap, we systematically mapped the global evidence base on the ecological, physical, economic, and social performance of NBS interventions related to coastal protection. We focused on active NBS interventions, such as restoring or creating habitat, adding structure, or modifying sediment in six shallow biogenic ecosystems: salt marsh, seagrass, kelp forest, mangrove, coral reef, and shellfish reef.

**Methods:**

We identified potentially relevant articles on the performance of NBS for coastal protection using predefined and tested search strategies across two indexing platforms, one bibliographic database, two open discovery citation indexes, one web-based search engine, and a novel literature discovery tool. We also searched 45 organizational websites for literature and solicited literature from 66 subject matter experts. Potentially relevant articles were deduplicated and then screened by title and abstract with assistance from a machine learning algorithm. Following title and abstract screening, we conducted full text screening, extracted relevant metadata into a predefined codebook, and analyzed the evidence base to determine the distribution and abundance of evidence and answer our research questions on NBS performance.

**Results:**

Our search captured > 37,000 articles, of which 252 met our eligibility criteria for relevance to NBS performance for coastal protection and were included in the systematic map. Evidence stemmed from 31 countries and increased from the 1980s through the 2020s. Active NBS interventions for coastal protection were most often implemented in salt marshes (45%), mangrove forests (26%), and shellfish reefs (20%), whereas there were fewer NBS studies in seagrass meadows (4%), coral reefs (4%), or kelp beds (< 1%). Performance evaluations of NBS were typically conducted using observational or experimental methods at local spatial scales and over short temporal scales (< 1 year to 5 years). Evidence clusters existed for several types of NBS interventions, including restoration and addition of structures (e.g., those consisting of artificial, hybrid, or natural materials), yet evidence gaps existed for NBS interventions like alteration of invasive species. Evaluations of NBS performance commonly focused on ecological (e.g., species and population, habitat, community) and physical (e.g., waves, sediment and morphology) outcomes, whereas pronounced evidence gaps existed for economic (e.g., living standards, capital) and social (e.g., basic infrastructure, health) outcomes.

**Conclusions:**

This systematic map highlights evidence clusters and evidence gaps related to the performance of active NBS interventions for coastal protection in shallow, biogenic ecosystems. The synthesized evidence base will help guide future research and management of NBS for coastal protection so that active interventions can be designed, sited, constructed, monitored, and adaptively managed to maximize co-benefits. Promising avenues for future research and management initiatives include implementing broad-scale spatial and temporal monitoring of NBS in multidisciplinary teams to examine not only ecological and physical outcomes but also economic and social outcomes, as well as conducting further synthesis on evidence clusters that may reveal measures of effect for specific NBS interventions. Since NBS can deliver multiple benefits, measuring a diverse suite of response variables, especially those related to ecosystem function, as well as social and economic responses, may help justify and improve societal benefits of NBS. Such an approach can help ensure that NBS can be strategically harnessed and managed to meet coastal protection goals and provide co-benefits for nature and people.

**Supplementary Information:**

The online version contains supplementary material available at 10.1186/s13750-024-00350-5.

## Background

Healthy coastal ecosystems provide services, ranging from food provisioning and carbon sequestration to nutrient cycling and water purification [2, 28, 55, 110]. These ecosystems, including salt marshes, seagrasses, mangroves, kelp forests, shellfish reefs, and coral reefs, also serve to buffer communities from coastal hazards by reducing physical impacts, such as shoreline erosion, wave energy [91], and storm surge [36]. For example, wave height can be reduced by salt marsh vegetation by 60% [59], fringing oyster reefs by 30–50% [107], and coral reefs by 84% [26]. The ability of coastal systems to dampen wave energy can reduce erosion [11, 80] and in some cases, trigger a shift from coastal erosion or shoreline retreat to accretion [56]. Attenuation of storm surge by mangrove forests [111] and marshes [1, 30] may also contribute to coastal protection by substantially decreasing the vulnerability of coastal communities.

Combined impacts from anthropogenic pressures, including climate change, threaten the capacity of coastal ecosystems to protect communities from hazards. Anthropogenic threats, including overexploitation, pollution, development, and habitat degradation, have triggered losses in habitat coverage across many coastal ecosystems, with global declines measuring 85% in oyster reefs [4], ~ 19–29% in seagrass meadows [17, 101], ~ 50% in coral reefs [18], 42% in salt marshes [31], 35% or higher in mangroves [34, 73, 98], and are also prevalent in kelp [19, 51]. Losses in habitat cover directly remove the structural components of the ecosystem (e.g., vegetation, reef substrate) that are largely responsible for coastal protection. Experimental evidence suggests that removing marsh vegetation limits the ability of marshes to reduce wave energy [59], and modeling efforts demonstrate linkages between coral reef loss and increases in wave energy [83]. As habitats are degraded or lost, their ability to provide ecosystem services, such as flood protection, is expected to decline [20, 90]. Mangrove deforestation in Myanmar, for example, decreased the total value of mangrove-associated ecosystem services by almost 30% over 14 years, of which almost 11% was attributed to a loss of coastal protection services [20].

With effects from climate change, including rising sea levels, changing precipitation patterns, intensifying storms, and increasing temperatures, the capacity of natural coastal ecosystems to protect communities can be overwhelmed or reduced, especially in systems already subjected to other sources of anthropogenic disturbance [91]. Projections under these extreme scenarios suggest that previously degraded coastal ecosystems will experience further changes, loss, and degradation [16, 29, 85, 109]. For example, mangroves may experience higher rates of erosion as wave heights increase with climate change [85], while coral reef regeneration may be impaired after storms when combined with additional stressors from anthropogenic activities [29]. When extreme events overcome the natural protection afforded by ecosystems, it can impose direct threats to and increase the vulnerability of coastal communities [61]. For instance, storm surge, which has already been responsible for almost half of the human fatalities from tropical cyclones in the United States from 1963 to 2012 [75], is expected to cause more fatalities as humans continue to migrate to coastal areas and the percentage of urban land at low elevations along the coast increases [37]. Additionally, coastal communities that are unwilling or unable to move may incur greater risks as flooding increases [58].

To improve coastal protection, resource managers, governments, local municipalities, tribal nations, military installations, non-governmental organizations, and private property owners are increasingly turning to nature-based solutions. Nature-based solutions (NBS) are broadly defined as “actions to protect, conserve, restore, and sustainably use and manage natural or modified terrestrial, freshwater, coastal, and marine ecosystems to address social, economic, and environmental challenges effectively and adaptively, while simultaneously providing human well-being, ecosystem services, resilience, and biodiversity benefits” [96]. Phrased more concisely, NBS are “actions that involve people working with nature, as part of nature, to address societal challenges, providing benefits for both human well-being and biodiversity” [81]. NBS is an umbrella term [66] that includes measures like green infrastructure, natural and nature-based features [5], nature-based infrastructure [89], natural infrastructure [24], nature-climate solutions [35], and ecosystem-based adaptation [13]. *Here, we focus on the subset of active NBS interventions used to improve coastal resilience to hazards by providing physical protective services, such as wave attenuation, flood reduction, and sediment stabilization.*

Active nature-based solutions for coastal protection can come in a variety of forms and may include the creation or restoration of a variety of ecosystems with or without the inclusion of engineered structural components. What these NBS techniques all have in common is the goal of providing some kind of physical protective service, such as reduced erosion and inundation, while also providing ecological co-benefits. Ecological co-benefits include, but are not limited to: increased biodiversity, improved water quality, and habitat enhancement, as well as the ability to adapt to and keep pace with stressors like sea level rise, that “gray” infrastructure (e.g., seawalls, bulkheads) either do not provide or exacerbate (e.g., block connectivity) [5, 6, 89]. Additional social benefits of NBS projects may include increased tourism [54], improvements in the aesthetic value of coastal habitats, and expanded access to cultural activities through environmental programs [14]. Economically, NBS often provide more cost-effective solutions for inundation protection, as they can eliminate typical maintenance costs and responsibilities associated with “gray” infrastructure [24, 86, 94], effectively preventing billions of dollars in flood-associated losses and repairs [76]. Although the economic and social benefits of NBS are often less thoroughly assessed than ecological benefits [87], primarily due to limited socio-economic data availability and difficulties in data collection [68], understanding the suite of benefits NBS provide can help coastal managers and decision-makers recognize the full potential of NBS projects for coastal protection [94].

Growing evidence that NBS can provide coastal protection (physical benefits) and other valuable ecological, economic, and social co-benefits if strategically designed, placed, constructed, and managed has spurred international efforts to broadly adopt NBS for protecting coastal communities and investments from threats of climate change and associated hazards [45, 47, 48], 95]. The United Nations and International Union for Conservation of Nature (IUCN), heralding the 2020s as the “Decade on Ecosystem Restoration,” called for approaches to reduce ecosystem degradation, one of which was nature-based solutions [97]. In the United States (US), this call has been met with landmark federal funding initiatives to boost the widespread use of NBS. Most recently, the US Infrastructure Investment and Jobs Act (IIJA, November 2021) allocated $47 billion for climate resilience projects, including billions of dollars for NBS to fortify coastal communities and improve resilience [38, 103], 105]. In Europe, the European Commission (EC) has also allocated funding to advance the development of NBS, including in coastal settings, and mainstream it internationally through the Horizon Europe research program (previously Horizon 2020) [21–23]. Some European countries also have their own national plans for NBS research and development. In Germany, the Climate and Transformation Fund will supply EU €4 billion until 2026, with the goal of improving ecosystem health and resilience [25]. NBS funding and initiatives are also prevalent in Latin American and Caribbean countries, including Mexico and Colombia [69] and Asian countries, including China [10] and Japan [93].

Despite recent increases in global implementation of NBS projects for coastal protection, substantial gaps in our understanding of NBS performance exist both broadly [82] and relative to coastal protection [78]. These gaps proliferate due to a lack of studies on the broader effectiveness of NBS, especially in coastal areas; a recent review of NBS effectiveness found that 13% of studies were conducted in coastal ecosystems, including coral reefs, mangroves, seagrass communities, and salt marshes—compared to forest (53%) and montane (19%) ecosystems [8]. Most NBS studies do not report on the full suite of NBS performance outcomes [8] because it is challenging to develop, as well as costly to measure, appropriate social and ecological [78, 82], as well as physical and economic [8, 82] performance standards. For example, measuring cost-effectiveness of NBS is difficult because the protection NBS affords depends on a variety of factors, such as the intensity and frequency of events an area experiences [82] or the time horizon over which costs are considered [24]. This is also the case for gray infrastructure, but with a key difference that NBS protective services are hypothesized to increase over time, while gray infrastructure protective services may decline [24]. NBS assessments are also challenging because performance metrics are highly technical, requiring specialized knowledge on projects that are often place-based (e.g., geomorphology) [8]. This is also true of gray infrastructure, but many modeling tools and design standards exist to help engineers design structures for specific levels of protection. Many NBS projects also do not budget for or require monitoring to ensure that projects meet expectations [32, 53, 67], reinforcing knowledge gaps. As interest and investments ramp up, the inability to address these gaps may undermine confidence in NBS implementation [8], including for coastal protection.

Surges in funding and subsequent construction of NBS for coastal protection, combined with the lack of NBS performance knowledge across geographies and conditions, have escalated the need to assess the performance of NBS for coastal protection. This study aims to identify, collate, and map the global evidence base on the ecological, physical, social, and economic performance of active NBS interventions used within the context of coastal protection in six biogenic, shallow (intertidal or subtidal) coastal ecosystems that face a variety of stressors and are among the most imperiled ecosystems on earth [33, 42]. The coastal ecosystems that we selected for inclusion in the systematic map are salt marsh, seagrass, kelp, mangrove, shellfish reef, and coral reef systems. The systematic map scope includes active NBS interventions for coastal protection, such as restoring or creating habitat, adding structure, or modifying sediment or morphology. The decisions to narrow the focus to six coastal ecosystems and active NBS interventions for coastal protection were made based on the primary research and management expertise of the systematic map team, as well as resource constraints. An improved understanding of NBS performance in shallow, biogenic coastal areas will help determine the breadth and depth of the knowledge base, highlighting both knowledge clusters and knowledge gaps.

### Stakeholder engagement

This systematic map was initiated by the National Oceanic and Atmospheric Administration (NOAA) National Centers for Coastal Ocean Science (NCCOS) to determine the state of knowledge regarding the performance of NBS for coastal resilience. The synthesis was motivated by a federally identified need to understand the evidence base surrounding NBS performance to help inform policy and management decisions about how to monitor NBS and when and where to implement NBS, as well as to identify where additional performance evaluations are warranted. Federal “team leads” for the synthesis effort developed a “core team” of federal researchers and academic scientists who study and implement NBS in estuarine and marine ecosystems. The core team helped refine the protocol scope, including research questions, inclusion criteria, and search strategy, and played key roles in compiling the systematic map. We also convened an “advisory team” of additional scientists and managers with expertise in NBS and coastal ecosystems to provide additional direction and feedback. The advisory team included scientists and managers from federal agencies, non-profits, and academia in the US. We engaged with the advisory team in one-on-one or small group virtual meetings and discussions. Several members of the advisory team helped refine the protocol by, for example, helping to represent the needs of their sectors, such as coastal managers. Discussions with the advisory team also helped refine our definitions for NBS and coastal protection, intervention typologies, outcome typologies, and data coding approach. Advisory team members also helped review and refine the systematic map findings and contributed additional sources during our call for literature. As neither our advisory group nor our core team include international scientists, we consulted additional scientists from countries outside of the US during the call for literature to help ensure that relevant international literature was incorporated into the systematic map and to reduce bias.

## Objective of the systematic map

The objective of this systematic map was to identify, collate, and map the global evidence base on the ecological, physical, social, and economic performance of active NBS interventions related to coastal protection in salt marsh, seagrass, kelp, mangrove, coral reef, and shellfish reef systems.

*The primary research question for the systematic map was:* What is the extent and distribution of evidence on the ecological, physical, social, and economic performance of active NBS interventions used in salt marsh, seagrass, kelp, mangrove, coral reef, and shellfish reef systems within the context of coastal protection? The elements of the primary question are:

- *Population:* Salt marsh, seagrass, kelp, mangrove, shellfish reef, or coral reef ecosystems where active NBS interventions are used.
- *Intervention: *Active NBS interventions established within the context of coastal protection. We used the term “active intervention” to mean the action of intentionally using, constructing, introducing, installing, or implementing NBS. We used the term NBS to describe NBS for coastal protection rather than NBS more broadly. Coastal protection must have been identified as a stated goal or measured outcome.
- *Comparator: *No comparator was required beyond presence of an active NBS intervention; however, studies that contained a comparator were also included. Examples of comparators are: presence vs. absence of NBS intervention, before vs. after NBS intervention, different types of NBS interventions (e.g., living shoreline vs. beneficial use), NBS monitored over time).
- *Outcome:* Ecological, physical, economic, or social performance outcomes evaluated following NBS interventions in the six coastal ecosystems.
- *Study type:* Experimental, observational, or modeling (using *in-situ* data) studies with quantitative or qualitative data on NBS performance outcomes

We also used the evidence base to answer multiple secondary questions about NBS performance:

- How does the extent and distribution of evidence on NBS performance differ across ecological (e.g., species and population, biological interactions, nutrient cycling), physical (e.g., water level, waves, sediment and morphology), social (e.g., human health, culture, safety and security), and economic (e.g., income, livelihoods, natural capital) outcomes?
- How does the extent and distribution of evidence on NBS performance differ by ecosystem type (e.g., salt marsh, mangrove, shellfish reef), NBS intervention type (e.g., system restoration or enhancement, system creation, structure addition), geographic location, and spatial scale?
- What approaches or methods are used to assess NBS performance? When is performance assessed relative to NBS implementation (e.g., < 1 year, 1–5 years, 5–10 years, > 10 years after construction)? What comparative approaches, if any, are used to assess NBS performance (e.g., presence vs. absence of NBS intervention, different types of NBS interventions, natural system vs. NBS intervention, no comparator)?
- Which coastal protection services (e.g., reduce shoreline erosion, attenuate wave energy, reduce inundation) do active NBS interventions seek to deliver?
- Which metrics (e.g., aboveground biomass, job creation) are used to assess NBS performance?

## Methods

The protocol for this systematic map was published in *Environmental Evidence* in May 2023 [71]. The systematic map adhered to the Collaboration of Environmental Evidence (CEE) Evidence Guidelines and Standards for Evidence Synthesis [12] and conformed to the RepOrting standards for Systematic Evidence Synthesis (ROSES) [41] (Additional File 1).

### Deviations

There were six deviations from the published protocol.

- First, because of the project timeline, funding availability, and staffing availability, we conducted the database searches prior to protocol publication in *Environmental Evidence.* We were fully prepared to modify the search string or the broader search strategy if any concerns had been raised or modifications recommended during peer-review of the protocol. This was a necessary yet acceptable risk, because before conducting the database searches, several NOAA librarians with expertise in search string development for evidence syntheses reviewed the search string and protocol manuscript. The NOAA librarians suggested several small changes to the search string, which we incorporated. We then submitted the protocol manuscript to NOAA internal review on February 2, 2023; this is required per NOAA policy. While the protocol was undergoing internal review and because of the aforementioned timeline constraints, as well as funding and staffing availability, we executed database searches from February 10, 2023 to February 20, 2023. During NOAA internal review, five scientists with expertise in NBS reviewed the manuscript; no additional modifications to the search strategy were recommended during NOAA internal review. After the protocol received final approval for submission through the NOAA-required internal review process, we submitted the protocol to *Environmental Evidence* on March 2, 2023. Peer-reviewers also recommended no modifications to the search string. The protocol was accepted on May 2, 2023 and published on May 22, 2023. Because no changes were required to the search string from either NOAA internal review nor *Environmental Evidence* review, we used results from the database search conducted in February 2023 for the map manuscript.
- Second, our protocol stated that we would hand-search reference sections of relevant reviews. Our database searches returned over 34,000 references and, because we searched 45 organizational websites, we did not have additional funding or staffing availability to hand-search.
- Third, our protocol stated that we would search the novel literature discovery tool Inciteful for 250 articles each from four categories: (1) similar papers, (2) most important relevant papers, (3) recent papers by the top 100 authors, and (4) most important recent papers. Instead, we searched for up to 1000 of the most relevant papers. We made this change in consultation with the Inciteful team to better capture the most pertinent papers based on our benchmarking articles.
- Fourth, our protocol stated that we would conduct single screening of articles during title and abstract screening. We decided to conduct double screening of the first 2300 articles to reduce bias. After double screening 2300 articles, we reverted to single screening as originally planned.
- Fifth, in our protocol we stated that we would use the Kappa statistic to evaluate inter-reviewer consistency. Instead, we decided to use percent agreeance to determine inter-reviewer consistency. We made this decision after reviewing previous systematic map protocols and accompanying systematic maps, where we found that many protocols intended to use the Kappa statistic but ultimately switched to using percent agreeance in their maps to assess inter-reviewer consistency.
- Sixth, we planned to search the United States Army Corps of Engineers as a single organizational website but decided to search two separate organizational websites that are part of the United States Army Corps of Engineers: (1) Engineer Research and Development Center and (2) Engineering with Nature because both contained a high number of potentially relevant articles.

### Search for articles

We conducted database searches from February 10, 2023 to February 20, 2023 in Web of Science, Scopus, Lens, Dimensions, ProQuest, Google Scholar, and Inciteful. We searched organization websites from May 3, 2024 to June 24, 2024, and we conducted a call for literature from May 6 to May 29, 2024. Searches were executed in English with a global geographic scope. The temporal scope of the search was from 1980 to present. This temporal scope was based upon a review of living shorelines, a common type of NBS, in which the earliest known study uncovered in the scoping review was from 1981 [87], suggesting that most studies on NBS with performance monitoring will be from 1980 to present.

#### Search string

We created the search string to align with key elements of the primary question, specifically the population and interventions. The population search string targeted eligible coastal ecosystems (i.e., salt marsh, shellfish reef, coral reef, mangrove, seagrass, kelp) and also included more general terms, like estuary and vegetation, used to refer to these ecosystems (Table 1, Additional File 2). The intervention search string was more complex because of the difficulty of searching for articles that reported on NBS intended to mitigate against coastal hazards and provide coastal protection benefits. We developed three substrings for the intervention string: (1) NBS, (2) hazards, and (3) mitigation (Table 1, Additional File 2). Both hazards and mitigation substrings helped identify papers focused on coastal protection. We did not develop a search string for outcomes because we wanted to cast a broad net across the range of possible outcomes in ecological, physical, social, and economic categories. Web of Science Core Collection was used to develop and test all search strings. The population and intervention search strings (Table 1) were employed together in different combinations to capture particular types of articles (Table 2). Additionally, since search strings were developed with Web of Science syntax, we modified search strings for the particular search platform to meet platform-specific syntax requirements. See the protocol [71] for additional details on search string development and testing. Final search strings are documented in Additional File 2.

**Table 1:** Search substrings created for population and interventions

PIO criteria	Concept	Substring (Web of Science syntax)
Population	Coastal ecosystems	oyster* OR mussel* OR bivalve* OR shell* OR cultch* OR coral* OR reef* OR marsh* OR saltmarsh* OR wetland* OR estuar* OR kelp OR seaweed* OR seagrass* OR "sea grass*" OR mangrove* OR swamp* OR mangal* OR "aquatic plant*" OR vegetation
Intervention	NBS	"Nature based solution*" OR "nature based strateg*" OR "nature based defen$e*" OR "nature based protection*" OR "nature based coastal" OR "nature based shoreline*" OR "nature based mitigation" OR "nature based infrastructure" OR "hybrid infrastructure" OR "hybrid technique*" OR "natural climate solution*" OR "natural infrastructure" OR "eco* engineer*" OR "ecosystem friendly engineering" OR bioengineer* OR "blue engineering" OR "building with nature" OR "engineering with nature" OR "working with nature" OR "nature derived solution*" OR "nature based feature*" OR "nature inspired solution*" OR "nature inclusive design*" OR "nature inspired design*" OR "nature derived design*" OR "soft protection strateg*" OR "soft shoreline*" OR "coastal adaptation*" OR "ecosystem* based adaptation*" OR "ecosystem* based measure*" OR "ecosystem* based mitigation" OR "disaster risk reduction" OR "living shoreline*" OR "coastal defen$e*" OR "natural barrier*" OR bioshield* OR "coastal protection" OR "protect* coast*" OR "shoreline protection*" OR "blue infrastructure" OR "soft defen$e*" OR "shoreline defen$e*" OR "managed realignment" OR "ecosystem based disaster risk reduction" OR "coastal resilienc*" OR "shoreline resilienc*" OR "restor* ecosystem* function*"
Intervention	Hazards (coastal protection)	"Coastal hazard*" OR "extreme weather" OR "extreme event*" OR "severe storm*" OR tsunami* OR typhoon* OR cyclon* OR hurricane* OR "tropical storm*" OR "storm surge*" OR monsoon* OR northeaster* OR nor'easter OR "sea level*" OR "high wind" OR "wave action”
Intervention	Mitigation (coastal protection)	Reduc* OR mitigat* OR protect* OR dissipat* OR dampen* OR attenuat* OR stabili$* OR trap* OR buffer* OR armour* OR armor* OR barrier* OR accret* OR adapt* OR breakwater*) OR AB = (reduc* OR mitigat* OR protect* OR dissipat* OR dampen* OR attenuat* OR stabiliz* OR trap* OR buffer* OR armour* OR armor* OR barrier* OR accret* OR adapt* OR breakwater*)) AND (TI = (hazard* OR erosion OR erod* OR flood* OR "storm surge*" OR wave* OR soil OR sediment* OR substrat* OR shoreline*
Intervention	Restoration	Construct* OR plant* OR install* OR restor* OR enhance* OR creat* OR retrofit*

Astericks (*) are wildcards

Substrings are in Web of Science Syntax. These strings were used for combined title OR abstract searching

**Table 2:** Search string combinations employed to capture articles on NBS for coastal protection

String combination	Search designed for
NBS AND Population	Articles focused on NBS concepts from target coastal ecosystems
NBS AND Mitigation	Articles focused on NBS concepts and coastal mitigation actions that do not explicitly mention target ecosystems in title or abstract
NBS AND Hazards	Articles focused on NBS concepts and coastal hazards that do not explicitly mention target ecosystems in title or abstract
Population AND Mitigation AND Hazards	Articles focused on coastal ecosystems and hazards and mitigations that do not explicitly use NBS or related terms in title or abstract
Population AND Mitigation AND Restoration	Articles focused on coastal ecosystems and mitigations that do not explicitly use NBS or related terms in the title or abstract but do use terms related to habitat restoration and creation

#### Comprehensiveness of the search

We identified 55 benchmarking articles to test our search string against (Additional File 3). These articles were sourced from subject matter experts, as well as from Smith et al. [87], a recent scoping review of living shorelines. Of the 55 benchmarking articles, 52 were indexed in Web of Science Core Collection. We conducted five rounds of testing until our search string captured all 52 articles indexed within Web of Science Core Collection. The remaining three articles were discovered in other search platforms. See the protocol for additional benchmarking details [71].

#### Indexing platforms

The indexing platform Web of Science (WOS) Core Collection was searched with the following specifications:

- Indexes: SCI-Expanded (1980–present); SSCI (1980–present); CPCI-S (1990–present); CPCI-SSH (1990–present); ESCI (2018–present)
- Document type: Article, Proceeding Paper, Early Access, Data paper
- Year: 1980–present
- Subscription: Duke University
- Date search executed: February 10, 2023

The indexing platform Scopus was also searched as follows:

- Year: 1980–present
- Subscription: Duke University
- Date search executed: February 10, 2023

#### Bibliographic databases

The bibliographic database ProQuest Earth, Atmospheric and Aquatic Sciences collection was searched using the following specifications:

- Indexes: Aquatic Sciences and Fisheries Abstracts; Meteorological and Geoastrophysical Abstracts; Earth, Atmospheric, & Aquatic Sciences Database
- Source type: Scholarly Journals, Dissertations & Theses, Conference Papers & Proceedings, Reports
- Year: 1980–present
- Subscription: NOAA
- Date search executed: February 13, 2023

#### Open discovery citation indexes

The open discovery citation index LENS was searched as follows:

- Indexes: CORE; Crossref; PubMed; Microsoft Academic
- Document type: Journal Article, Conference Proceeding Article, Conference Proceedings, Dissertation, Report
- Year: 1980–present
- Subscription: N/A
- Date search executed: February 10, 2023

The open discovery citation index Dimensions was search as follows:

- Document type: Article, Proceeding
- Year: 1980–present
- Subscription: NOAA
- Date search executed: February 10, 2023

#### Web-based search engine

The web-based search engine Google Scholar was searched using Publish or Perish version 8 [43]. The simplified search string used for Google Scholar due to reduced capabilities to use Boolean logic was:

("nature based solutions" OR "nature based infrastructure") AND ("salt marsh" OR mangrove OR kelp OR seagrass OR coral OR shellfish OR oyster) AND ("coastal protection")

The search was performed on titles for up to 1000 articles on February 10, 2023, following recommendations for how to use Google Scholar in evidence syntheses [40].

#### Novel literature discovery tool

The novel literature discovery tool Inciteful (https://inciteful.xyz/; [102]) was searched for up to 1000 most relevant papers on February 20, 2023. We seeded the Inciteful search using a.RIS file of benchmarking articles (Additional File 3).

#### Organizational databases and websites

Forty-five organizational databases and websites were searched for relevant gray literature from May 3, 2024 to June 24, 2024 (Additional File 4):

- Asian Development Bank: https://www.adb.org/
- Australian Government Department of Climate Change, Energy, the Environment, and Water: https://www.dcceew.gov.au/
- Billion Oyster Projecthttps://www.billionoysterproject.org/
- Caribbean Natural Resources Institute: https://hub.canari.org/
- Climate Resilient by Nature: https://www.climateresilientbynature.com/
- ClimateLinks: https://www.climatelinks.org/
- Commonwealth Scientific and Industrial Research Organisation: https://www.csiro.au/
- Conservation International: https://www.conservation.org/
- UK Government Foreign, Commonwealth & Development Office (formerly called: UK Government Department for International Development): https://www.gov.uk/
- USAID Development Experience Clearinghouse: https://www.usaid.gov/
- Deutsche Gesellschaft fur Internationale Zusammenarbeit: https://www.giz.de/
- Environmental and Energy Study Institute: https://www.eesi.org/
- Environmental Defense Fund: https://www.edf.org/
- European Union / Commission: https://op.europa.eu/
- Global Facility for Disaster Reduction and Recovery: https://www.gfdrr.org/
- Global Mangrove Alliance: https://www.mangrovealliance.org/
- Global Program on Nature-Based Solutions for Climate Resilience: https://naturebasedsolutions.org/
- iied Publications Library: https://www.iied.org/
- International Monetary Fund: https://www.imf.org/
- International Union for Conservation of Nature: https://www.iucn.org/
- National Fish and Wildlife Foundation: https://www.nfwf.org/
- National Oceanic and Atmospheric Administration: https://www.noaa.gov/
- National Science Foundation: https://www.nsf.gov/
- Oxford Nature Based Solutions Initiative: https://www.naturebasedsolutionsinitiative.org/
- rare: https://rare.org/
- Resources for the Future: https://www.rff.org/
- The Nature Conservancy: https://www.nature.org/
- United Nations Decade on Restoration: https://www.decadeonrestoration.org/
- United Nations Development Programme: https://www.undp.org/
- United Nations Environment Programme: https://www.unep.org/
- United Nations Environment Programme World Conservation Monitoring Center: https://resources.unep-wcmc.org/
- United States Army Corps of Engineers—Engineer Research and Development Center: https://erdc-library.erdc.dren.mil
- United States Army Corps of Engineers—Engineering with Nature: https://ewn.erdc.dren.mil/
- United States Climate Resilience Toolkit: https://toolkit.climate.gov/
- United States Department of Transportation: https://www.transportation.gov/
- United States Environmental Protection Agency: https://www.epa.gov/
- United States Fish and Wildlife Service: https://www.fws.gov/
- United States Geological Survey: https://www.usgs.gov/
- University of Georgia Institute for Resilient Infrastructure Systems: https://iris.uga.edu/
- Wetlands International: https://www.wetlands.org/
- Wildlife Conservation Society: https://library.wcs.org/
- World Agroforestry Center: https://www.worldagroforestry.org/
- World Bank: https://www.worldbank.org/
- World Resources Institute: https://www.wri.org/
- World Wildlife Fund: https://www.worldwildlife.org/

Most organizational databases and websites did not allow Boolean searches so the detailed search strings (Table 2) were adapted to match the search functionality of each website. The first 100 search results from each organizational website were screened in situ*.*

### Call for literature

We conducted a call for literature from May 6–May 29, 2024. The call for literature was distributed via email to 66 community members and one broader listserv to request gray literature. The community members included experts in NBS from Australia, Belgium, Brazil, Canada, Chile, China, Netherlands, New Zealand, South Africa, United Kingdom, United States, and Singapore. If an expert provided more than 100 references, then we screened the first 100 results in situ.

#### Assembling and managing search results

Search results from indexing platforms, bibliographic databases, open discovery citation indexes, Google Scholar via Harzings, and the novel literature discovery tool were exported as separate.RIS files. These.RIS files were imported into R version 4.2.2 [74], assigned a source (e.g., Web of Science, Scopus) and deduplicated using CiteSource [77] first within a database (e.g., WoS) and then across databases (e.g., WoS, LENS, etc.). Following deduplication in CiteSource, references were exported as one combined.RIS file from R. The combined.RIS file was imported to EndNote version 21.2 [92]. Within EndNote, we conducted manual deduplication following the workflow from McKeown and Mir [57]. Specifically, we merged duplicates but ensured that the record ID of the discarded duplicate was collated to the record ID of the retained duplicate to allow article tracking. Articles discovered during organizational website searches and the call for literature were deduplicated against the full search results (e.g., indexing platforms, bibliographic databases) during in situ screening; these articles were added to the final map.RIS file.

### Article screening and study eligibility criteria

#### Screening process

Articles were screened first by title and abstract and second by full text. Screening at the title and abstract level was conducted in Swift Active Screener [44]. Swift Active Screener uses active learning to rank publications in order of relevance based on screener feedback so that relevant publications can be prioritized for screening. The software presents a running estimate of the percentage of relevant references that have been screened, referred to as the “recall rate.” We conducted screening in Swift until the software’s “recall rate” reached 95% [44].

Prior to commencing title and abstract screening, each screener was trained using a workflow from Paxton et al. [70]. First, screeners attended a training session with information on the project background and instructions on how to screen and on how to use Swift Active Screener. During the training session, 10 articles were screened together. Following the training session, each screener screened 10 articles independently, responses were compared, and inconsistencies were discussed and resolved. Next, each screener screened an additional 30 articles independently, and the results were again compared, and inconsistencies discussed and resolved. We then determined inter-reviewer consistency on a set of 100 randomly selected articles. We used percent agreeance to evaluate consistency; each pair achieved 95% or higher consistency. Following these training activities, screeners were authorized to begin screening in earnest.

Eleven screeners conducted title and abstract screening in Swift Active Screener. During title and abstract screening, we started by double screening articles in Swift Active Screener. If screeners’ decisions to include or exclude an article differed, the inconsistency was discussed and resolved. We double screened 2300 articles, at which point the recall rate was 38.7% in Swift Active Screener. These double screened articles met our criteria for quality assurance and quality control. After double screening 2300 articles, we switched to single screening for the remainder of title and abstract screening.

Seven screeners conducted full text screening. Full text screening was conducted using an online spreadsheet (see details below) so that our multi-institutional team could screen individual articles simultaneously. Full texts were stored and accessed in EndNote. Some screeners were not able to access EndNote, so the full texts they were assigned were stored and accessed via Google Drive. All screeners except one had also participated in title and abstract screening so were familiar with the screening eligibility criteria. Screeners reviewed instructions on how to screen articles at the full text level. Any uncertainties during full text screening were discussed and resolved with at least one reviewer, and in several instances with the whole team.

The screener who had not participated in full text screening attended a virtual meeting with the project lead (ABP), where they were introduced to the systematic mapping process, project goals, and screening process. After this introductory meeting, the screener reviewed the published protocol, full text screening instructions, and full text screening spreadsheet. The screener then attended another meeting with the project lead to discuss questions and verbally review the full text screening process, eligibility criteria, and data coding. The screener was assigned four training articles to screen and code. After screening and coding the training articles, the screener met with the team lead to compare screening decisions and data coding and discuss and resolve any inconsistencies. After this meeting, the screener was cleared to begin full text screening in earnest.

We conducted quality assurance and quality control by rescreening 33 articles (5%), which were randomly selecting using a custom R code. This number of articles was 5% of the number of articles for which full texts were available (n = 662); it did not include articles discovered from organization websites or the call for literature. There was one article that was originally excluded that was changed to included because it contained runnels, which was a type of active NBS intervention characterized by tidal change extensions [100]. For both screening stages, if a screener is an author of an article, they were not permitted to screen the article nor code metadata extraction.

#### Eligibility criteria

Articles were screened using the following eligibility criteria:

- *Relevant population(s):* Relevant populations were six types of shallow coastal ecosystems: salt marsh, seagrass, kelp, mangrove, shellfish reef, and coral reef. These systems could be either existing (e.g., where NBS is constructed in an existing salt marsh or near an existing salt marsh) or created (e.g., NBS constructed to create salt marsh in an area where salt marsh is currently nonexistent). We defined salt marsh as estuarine or brackish marsh, not freshwater. Shellfish reefs were defined as oyster, mussel, or other reef-forming bivalves. Coral reefs were defined as shallow systems, not deep or mesophotic. Other coastal systems, such as dunes, beaches, rocky reefs, and maritime forests were excluded because they were beyond the scope of the study. Deep sea, freshwater, subterranean, and terrestrial systems were excluded. If, however, a study includes one or more of the six eligible ecosystems and one or more of the excluded ecosystems, the study passed the population screening. For instance, if a study reported on kelp and rocky reefs, the study would be included since it reports on one of the six target ecosystems (kelp), even though it also includes content on an excluded system (rocky reef). If there was added structure of human-made, hybrid, or natural origin, it was included if it was installed in OR to form or restore one of the six relevant ecosystems. For example, if an article reported on concrete modules installed on a coral reef OR concrete modules installed on sand bottom to restore a coral reef, it was included.
- *Relevant intervention(s):* Relevant interventions used active NBS within the context of coastal protection (Table 3). To be active interventions, NBS must be used, installed, constructed, or implemented by humans, such as through actions like restoring or creating habitat, adding structure, retrofitting or modifying structure, modifying sediment or morphology, or removing or adding invasive species. To be related to coastal protection, NBS interventions must *either* have a stated goal or evaluated outcome of coastal protection, or both a stated goal and evaluated outcome. To meet the “stated goal” provision, NBS must be stated to have a goal, aim, or intent of coastal protection related to waves, current, wind, water level, storm surge, sediment, or morphology. To meet the evaluated outcome provision, NBS must be evaluated for physical outcomes (any directionality—positive, negative, neutral) related to waves, current, wind, water level, storm surge, sediment, or morphology. Passive NBS interventions, such as those involving protecting, conserving, or managing coastal ecosystems were excluded. NBS interventions that were designed, planned, or sited but not implemented were excluded. Existing ecosystems without an NBS intervention (e.g., a salt marsh that inherently provides coastal protection) were excluded. See Additional File 5 for intervention typologies.
- *Relevant comparator(s):* No comparator was required because studies that includes an active NBS intervention related to coastal protection were included. Studies that contained a temporal or spatial comparator, though, were included. Temporal comparators included those that report NBS performance over time gleaned from long-term monitoring, experimental observations, or before vs. after NBS intervention. Spatial comparators included those that reported NBS performance over space gleaned from locations with or without NBS interventions, or locations with different types of NBS interventions.
- *Relevant outcome(s):* Ecological, physical, economic, or social performance outcomes of NBS that are measured, observed, or modeled. See Additional File 6 for outcome typologies. Studies that did not report performance within one of the four main categories (ecological, physical, social, economic) were excluded.
- *Relevant study type(s):* Observational (e.g., monitoring, assessment) or experimental studies from peer-reviewed publications and gray literature conducted in situ. Lab studies conducted in greenhouses, flumes, or similar were excluded. Modeling studies were excluded unless they used in situ field data. Reviews, theoretical studies, commentaries, editorials, opinions, or perspectives were excluded.

**Table 3:** Typology of NBS interventions

Category	Definition	Example
System restoration, enhancement, or rehabilitation	Intervention restoring, enhancing, or rehabilitating natural habitat or systems and associated services	Restoration; habitat enhancement; rehabilitation; repopulation; these are for when a habitat currently exists that is improved upon or expanded; code as creation if it is a new system
System creation	Intervention creating system in place of a naturally occurring one	Creation of salt marsh on urban shoreline; creation of oyster reef on mud flats
Structure addition of human origin (artificial)	Intervention adding artificial, engineered, or designed structure to an existing ecosystem	Reef modules; reef ball; reef dome; mini domes; oyster ball; oyster catcher; oyster castle; biorock; artificial seagrass
Structure addition of natural origin	Intervention adding structure of natural origin (even if from a non-coastal ecosystem) to an existing ecosystem	Rock; oyster cultch; marsh mat; marsh mattress; marsh pillow; marsh blanket; biodegradable element; coir mats; logs; trees; rock sill
Structure addition of hybrid origin (both artificial and natural)	Intervention adding structure of mixed natural and human origin to an existing ecosystem	Gabion basket; shade net; biorock; shell bag; oyster bag; concrete / lattice marsh mattresses
Retrofitting or removing gray infrastructure	Intervention retrofitting, modifying, or removing gray infrastructure	Retrofitting, modifying, or removing: culvert, seawall, bulkhead, berm, dike, levee, tide gate
Sediment stabilization, removal, or placement	Interventions stabilizing, removing, or placing sediment in a system	Sediment stabilization; sediment trapping; sediment or fill removal; beneficial use; beneficial reuse; thin-layer placement or application
Morphology modification	Interventions modifying the morphology of a system through changing the topography or morphology	Grading; regrading; terracing; platforming; island building
Invasive species modification	Interventions involving modification of invasive species through removal or addition	Invasive species removal; invasive species addition

Typologies adapted from [5–9, 46, 82]; see additional details in Additional File 5

#### Study validity assessment

Because this is a systematic map meant to compile a broad evidence base, we did not systematically assess the study validity through conducting critical appraisals as is typical in systematic reviews. Attributes extracted during data coding could be used for future assessments of study validity.

#### Data coding strategy

We entered metadata from studies that passed full text screening into an online ‘data coding’ spreadsheet (Google Spreadsheets), where each study corresponded to a spreadsheet row. We extracted and coded attributes for bibliographic information, population, intervention, study type, comparator, and outcomes in the data coding spreadsheet. Associated intervention and outcome typologies (Additional File 5; Additional File 6) were also extracted and coded in the spreadsheet. Details of each attribute were specified in a codebook (Additional File 7) that also included instructions for data entry and levels of categorical variables that could be selected from dropdowns. Some categorical variables could have multiple selections (e.g., a population that included both seagrass and mangrove), and attributes that could have multiple selections were also identified and the multi-selection function was scripted using Google Apps Script extension. We did not contact authors to request missing information, but rather used information contained in the full text or supporting information.

Seven data coders were trained on metadata extraction during the full-text training. We did not conduct double extraction because of the high number of articles that required coding. Rather, when an article was encountered for which a data coder was uncertain whether the article should be included or excluded, we discussed these articles in small groups to resolve the uncertainty. Spot checks were conducted for 100% of the included coded articles discovered during the full database searches, call for literature, and organizational website searches. During spot checks, which were conducted by two data coders, we checked for and corrected dissimilarities in spelling, deviations from pre-defined factor levels, ambiguous metadata, and any other uncertainties. We then exported coded data from the online spreadsheet as a.csv file that we imported to R for analysis and visualization.

#### Data mapping method

We completed the ROSES flow diagram [39] to provide an overview of the systematic map process. We analyzed coded data in R version 4.2.2 [74] to answer the primary and secondary research questions. Specifically, we investigated and visualized patterns in the distribution and abundance of evidence surrounding NBS performance by: descriptive information (publication type, publication date, geography), ecosystem type, NBS intervention type, coastal protection context, study type (e.g., spatial scale, comparator type, cost reported). We also visualized evidence on NBS performance outcomes, across ecological (e.g., species and population, biological interactions, nutrient cycling), physical (e.g., water level, waves, sediment and morphology), social (e.g., human health, culture, safety and security), and economic (e.g., income, livelihoods, natural capital) categories. We identified evidence clusters and gaps using heat maps based on matrices of the number of studies for cross-tabulated attributes (e.g., interventions versus outcomes). Visualizations were created using the R package “ggplot2” [108].

## Results

### Systematic mapping process

**Fig. 1:**
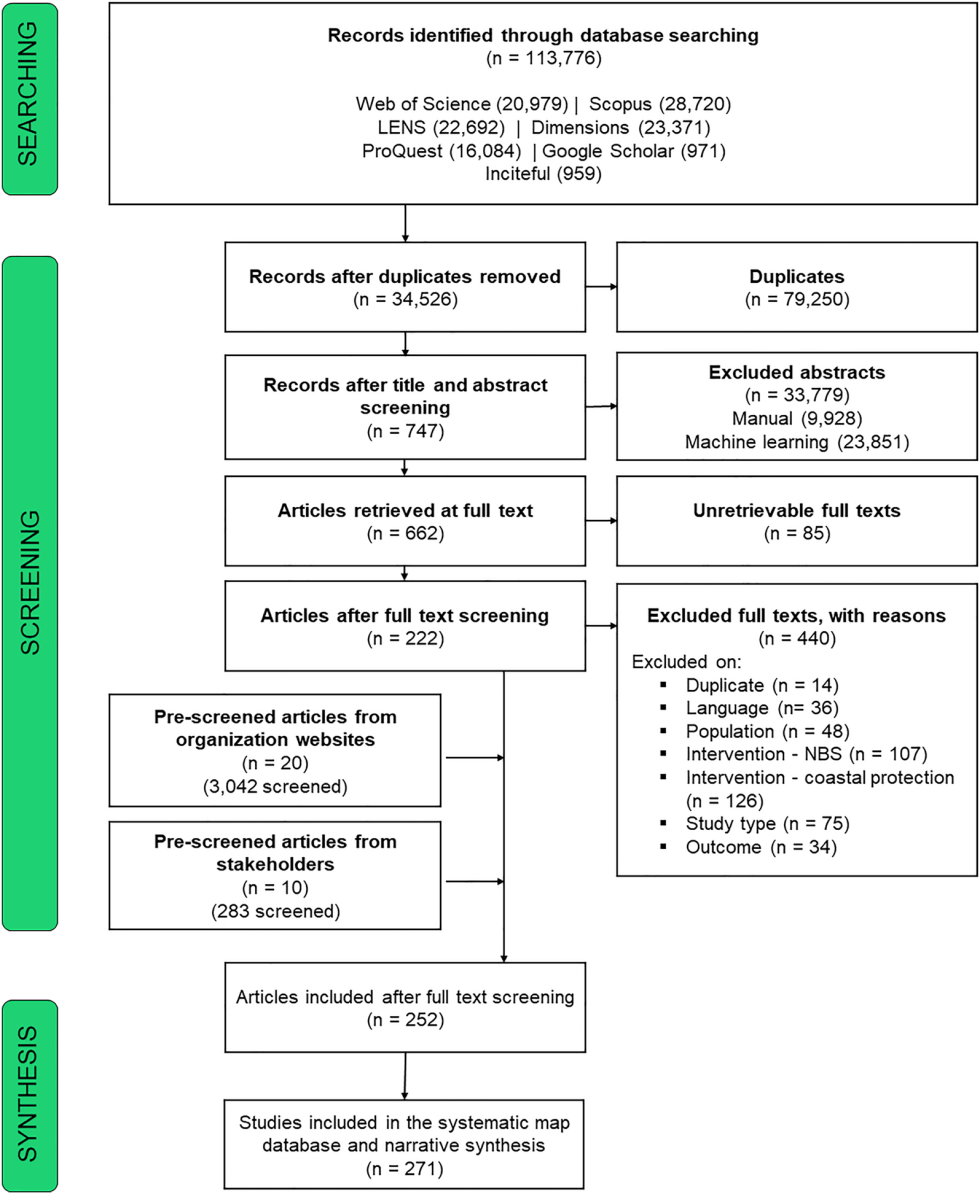
ROSES flowchart displaying the number of articles returned from initial search and included during subsequent stages of the systematic map process. Flowchart from Haddaway et al. [39]

The number articles returned during each stage in the systematic map process is reported in the ROSES flowchart (Fig. 1). Database searches identified 113,776 records. These database records included 28,720 from Scopus, 23,371 from Dimensions, 22,692 from LENS, 20,979 from Web of Science, and 16,084 from ProQuest. The novel search tool Inciteful returned 959 records, and Google Scholar yielded 959 records. Of the 113,776 database records, 79,250 were identified as duplicates. After duplicate removal, 34,526 records remained and were screened at the level of title and abstract. During title and abstract screening, 33,779 records were excluded; some of these records were excluded manually (n = 9928) whereas others were excluded using machine learning (23,851). After title and abstract screening, 747 articles remained.

Of the remaining 747 articles, full texts were retrievable for 662 articles (n = 85 unretrievable; Additional File 9 with exclusion reason “full text availability”). During full text screening, we excluded 440 articles and included 222 articles. Articles were excluded during full text screening because they did not contain an eligible intervention either for coastal protection context (n = 126) or NBS type (n = 107). Other articles were excluded because they were the improper study type (n = 75), an ineligible coastal ecosystem population (n = 49), or written in a non-English language (n = 36). Some articles were excluded because they did not report on outcomes (n = 34) or because they were duplicates (n = 14) that had been undetected previously because of slight differences in article metadata.

In addition to database searches, articles were discovered during organizational website searches and a call for stakeholder contributed literature. Organizational website searches returned 3,042 potentially relevant articles, of which 20 were included during in situ screening. Stakeholders provided 283 articles in response to the call for literature, of which 10 were included during in situ screening.

In total, 252 articles (n = 222 databases, n = 20 from organizational websites, n = 10 from stakeholder contributed literature) were included in the systematic map after full text screening; these 252 articles are included in the resulting systematic map database and narrative synthesis. These articles encompassed 271 studies. The ROSES reporting form is in Additional File 1. Additional File 8 contains the bibliography of included articles. Additional File 9 contains the bibliography of excluded articles and their exclusion rationales. Additional File 10 contains coded data for included articles.

### Descriptive information

In the descriptive information reported below, articles can appear in more than one category. For example, an article can have multiple coastal ecosystem population categories (seagrass, saltmarsh) or multiple NBS intervention types (structure addition, restoration). Thus, the total sample size can be greater than the total number of articles (n = 265). In several instances, an article contained multiple case studies characterized by separate NBS projects that were not compared. If an article contained two or more eligible case studies, each case study was coded separately, such that the total number of studies was 271.

#### Publication type

**Fig. 2:**
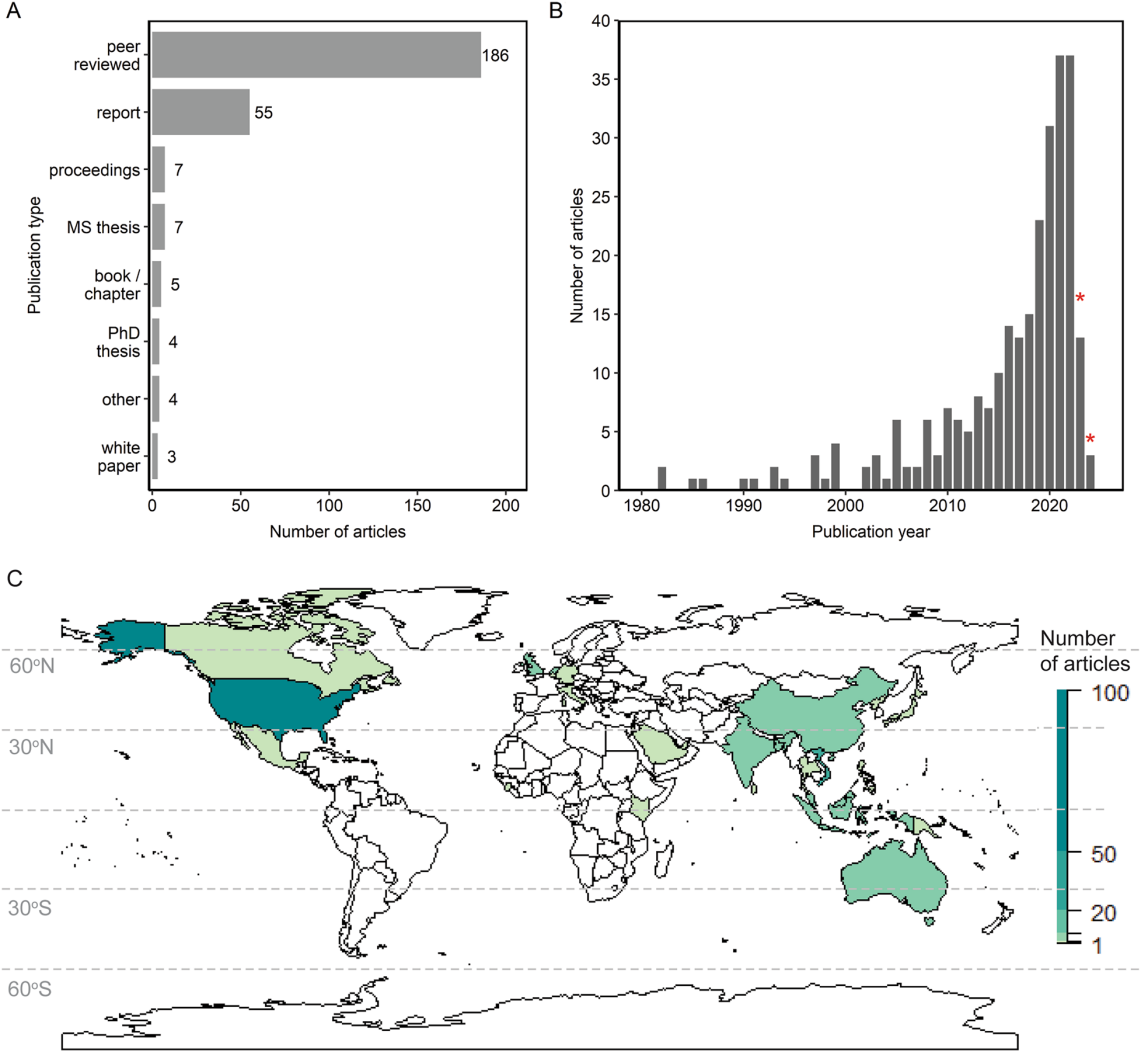
Number of articles by **a** publication type, **b** publication year, and **c** country. For (**b**), the red asterisks indicate partial years for when the search was conducted. In (**c**), countries in white have zero articles, and dashed lines indicate 30° latitude increments

Peer-reviewed publications (69%, n = 184) and reports (2%, n = 55) constituted the majority of articles in the systematic map (Fig. 2a). Several articles were MS theses (3%; n = 7), proceedings (3%; n = 7), books or book chapters (2%; n = 5), PhD theses (2%; n = 4), other (2%; n = 4), or white papers (1%; n = 3).

#### Publication year

The number of articles published per year increased from 1980 through the present (Fig. 2b). The earliest published articles were from 1982 (n = 2). There were four articles published in the 1980s, 13 articles during the 1990s, and 25 articles during the 2000s. During the 2010s, the number of articles increased substantially to 108. So far during the 2020s, there have been 121 published articles. Our search was executed during 2023, and several stakeholder-contributed articles stemmed from 2024. The year 2022, which was the last full year included in the search, had the highest number of published articles in a given year (n = 37).

#### Publication geography

Evidence on NBS performance arose from 33 countries (Fig. 2c; Table 4). The majority of evidence (54%) stemmed from the United States (n = 149). The other top countries represented in the evidence base were United Kingdom (n = 17), Vietnam (n = 16), China (n = 12), Indonesia (n = 10), Netherlands (n = 10), Australia (n = 7), and China (n = 7), which collectively accounted for an additional 26% of the evidence base.

**Table 4:** Geographic distribution of evidence (number of articles) per country

Country	Number of articles
United States	149
United Kingdom of Great Britain and Northern Ireland	17
Vietnam	15
China	12
Indonesia	10
Netherlands	10
Australia	7
Malaysia	6
Bangladesh	5
India	5
Singapore	4
Kenya	3
Mexico	3
Thailand	3
Canada	2
Italy	2
Japan	2
Philippines	2
Sri Lanka	2
United Arab Emirates	2
Belgium	1
Chile	1
Democratic People's Republic of Korea	1
Dominican Republic	1
Germany	1
Grenada	1
Israel	1
Maldives	1
Papua New Guinea	1
Portugal	1
Puerto Rico	1
Saudi Arabia	1
Sierra Leone	1

#### Ecosystem types

**Fig. 3:**
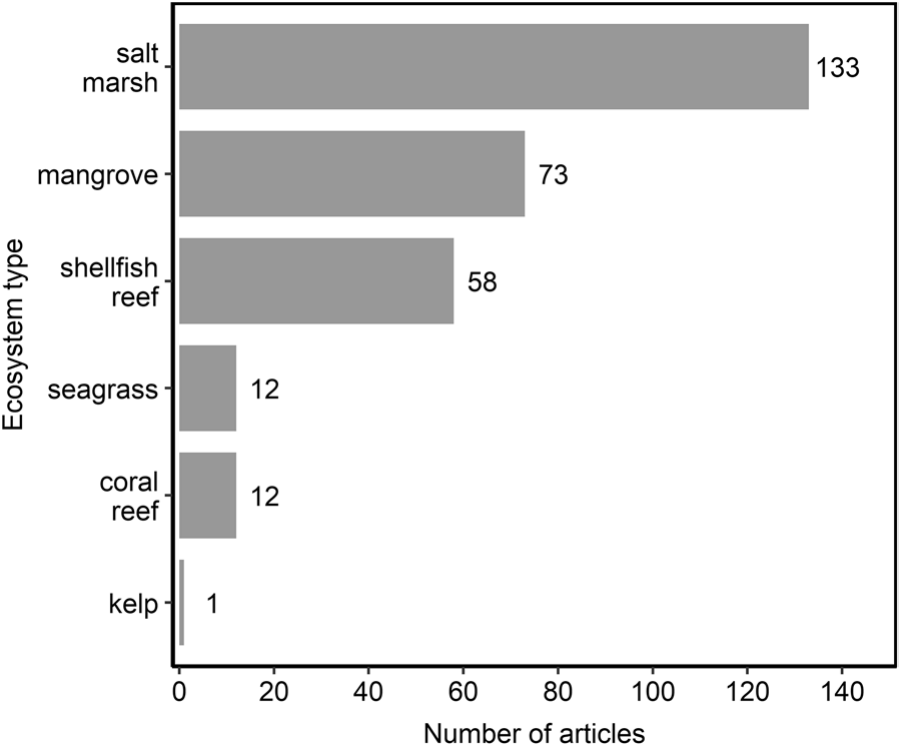
Number of articles by ecosystem type (population). Some articles contained more than one population so can appear in more than one category

Evidence existed for all six types of shallow coastal ecosystems that constituted relevant populations, although some ecosystem types had several orders of magnitude more evidence than others (Fig. 3). Salt marshes contained the most evidence on NBS performance (46%; n = 133), followed by mangroves (25%; n = 73), and shellfish reefs (20%; n = 58). Seagrass (4%; n = 12) and coral reefs (4%; n = 12) had the same amount of evidence as each other. Kelp (< 1%; n = 1) had the least amount of evidence.

#### Characteristics of nature-based solutions interventions

##### Types of NBS interventions

**Fig. 4:**
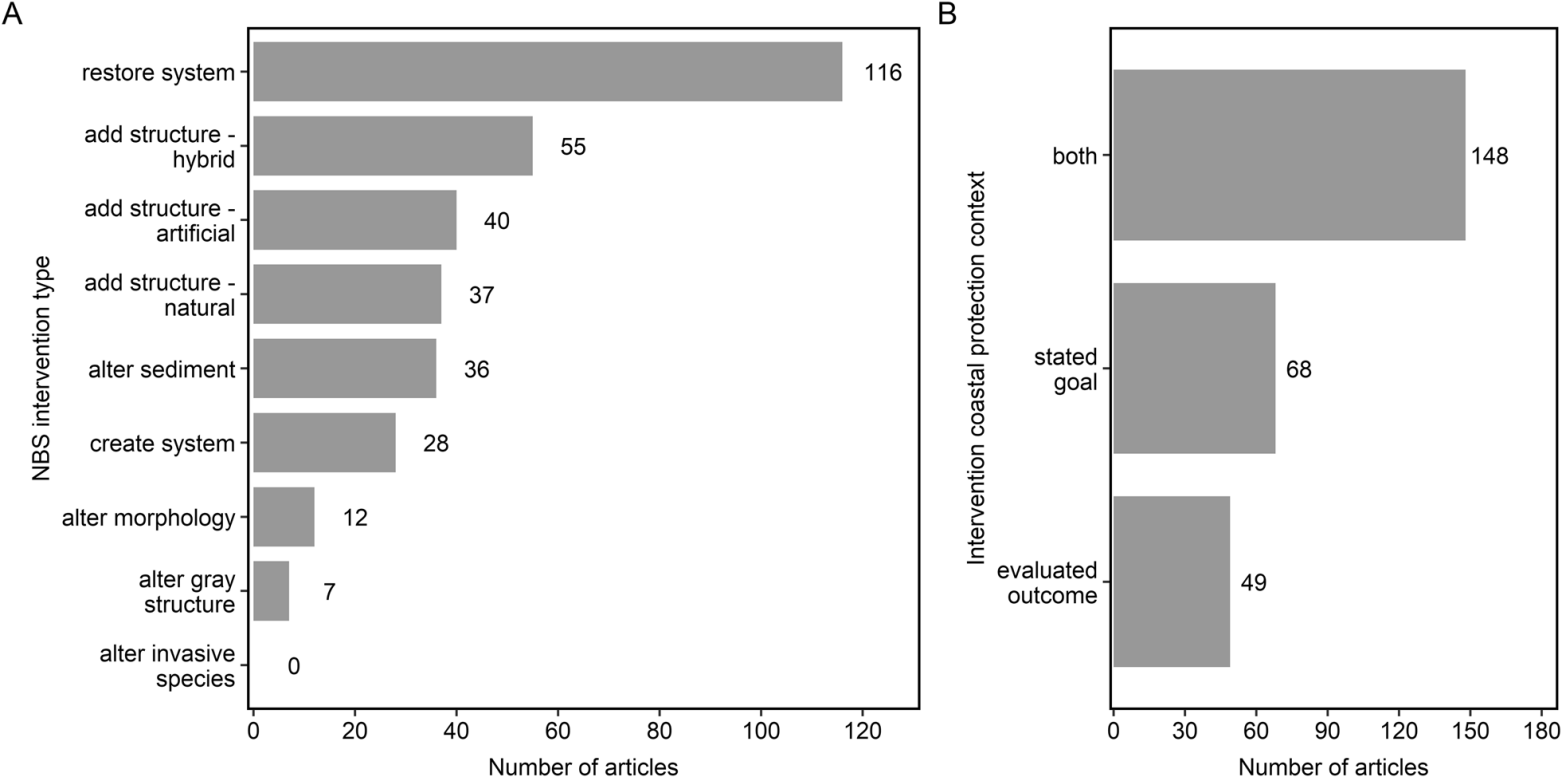
Number of articles by **a** NBS intervention type and **b** the coastal protection context of the NBS intervention. Some articles contained more than one intervention so can appear in more than one category

Most NBS interventions were ecosystem restoration, enhancement, or rehabilitation (35%; n = 116), such as salt marsh or shellfish reef restoration (Fig. 4a). Other common intervention types included adding structure (40%; n = 132) categorized as hybrid (17%; n = 55), artificial (12%; n = 40), or natural (11%; n = 37) to an existing ecosystem. Hybrid structures of mixed natural and human origin ranged from bagged oysters and artificial seagrass mats to riprap. Artificial structures encompassed reef modules like Reef Balls^®^, trapezoidal units, and Oyster Castles^®^, as well as coastal armoring structures like retrofitted seawalls. Natural structures often included rocks or sediment fences, such as those constructed from recycled Christmas trees. Other intervention types included sediment stabilization, placement, or removal (11%; n = 36) through beneficial re-use or thin-layer application sediment. Other studies used ecosystem creation (9%; n = 28), where a system was created in place of a naturally occurring one. Some studies also used morphology modification (4%; n = 12) like installation of runnels or managed realignment. Several studies included retrofitting or removal of gray infrastructure (2%; n = 7). No studies contained interventions involving modification of invasive species.

##### Policy names for NBS interventions

Most NBS interventions were referred to as “restoration” (n = 104) or “living shorelines” (n = 56). The term “nature-based solutions” was used in 33 cases. Other terms included “rehabilitation” (n = 20), “managed realignment” (n = 13), “nature-based coastal defense” (n = 11), “artificial reef” (n = 8), “natural infrastructure” (n = 7), “natural and nature-based feature” (NNBF; n = 4), “ecological engineering” (n = 4), and “green engineering” (n = 2). There was one instance each of the following terms: ecosystem-based adaptation, ecosystem-based disaster risk reduction, climate adaptation service, green infrastructure, and blue engineering. There were no instances with the following terms: community-based adaptation, ecosystem-based mitigation, or blue infrastructure.

##### Coastal protection context of NBS interventions

Most NBS interventions within the evidence base contained both a stated goal and an evaluated outcome related to coastal protection (56%; n = 148; Fig. 4b). Other studies only had a stated goal of coastal protection (26%; n = 68), whereas some only had an evaluated outcome related to coastal protection (19%; n = 49). When coastal protection was a stated goal, it was often to control erosion, reduce wave energy, increase shoreline or habitat elevation, or defend against flooding. In many cases, the goal of the NBS intervention was to more generally provide “coastal protection,” and specific aspects of the goal were not identified.

#### Study types

**Fig. 5:**
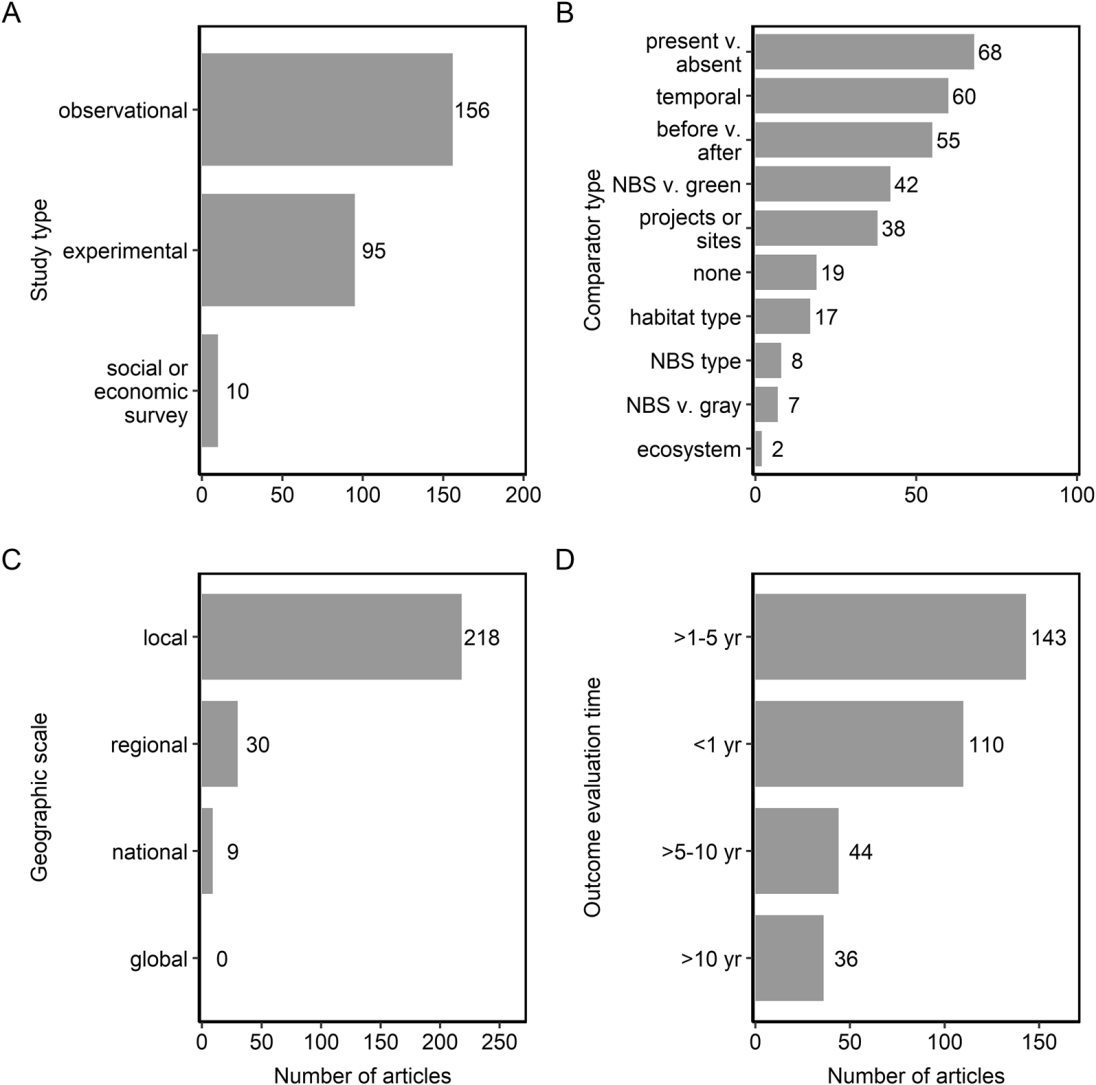
Number of articles by **a** study type, **b** comparator type, **c** geographic scale, and **d** length of outcome evaluation time in years. Some articles contained more than one study type or outcome evaluation time length so can appear in more than one category

The majority of the evidence base stemmed from observational studies (60%; n = 156; Fig. 5a). Other study types reflected in the evidence base include experimental studies (36%; n = 95) and social or economic surveys (4%; n = 10). Some studies did not have comparators (6%; n = 19; Fig. 5b). Of the studies with comparators, most compared performance of NBS presence versus absence (22%; n = 68), monitored NBS performance over time (19%; n = 60), or compared performance before versus after NBS interventions (17%; n = 55). Other studies compared performance of NBS interventions to natural systems (e.g., green; 13%; n = 42) or assessed the performance of NBS across different projects or sites (12%; n = 38). Some studies compared NBS performance: between or among different habitat types (5%; n = 17), across different NBS types (2%; n = 8), to gray infrastructure (2%; n = 7), or across different ecosystems (< 1%; n = 2).

The majority of evidence stemmed from studies conducted at local spatial scales (85%; n = 218; Fig. 5c). Fewer studies examined NBS performance across regional scales (12%; n = 30) or national scales (4%; n = 9). No studies examined NBS performance at global scales. While 25 studies (9%) did report the cost of NBS interventions, most studies did not report on the cost of NBS interventions (91%; n = 243).

#### Ecological, physical, economic, and social outcomes examined

**Fig. 6:**
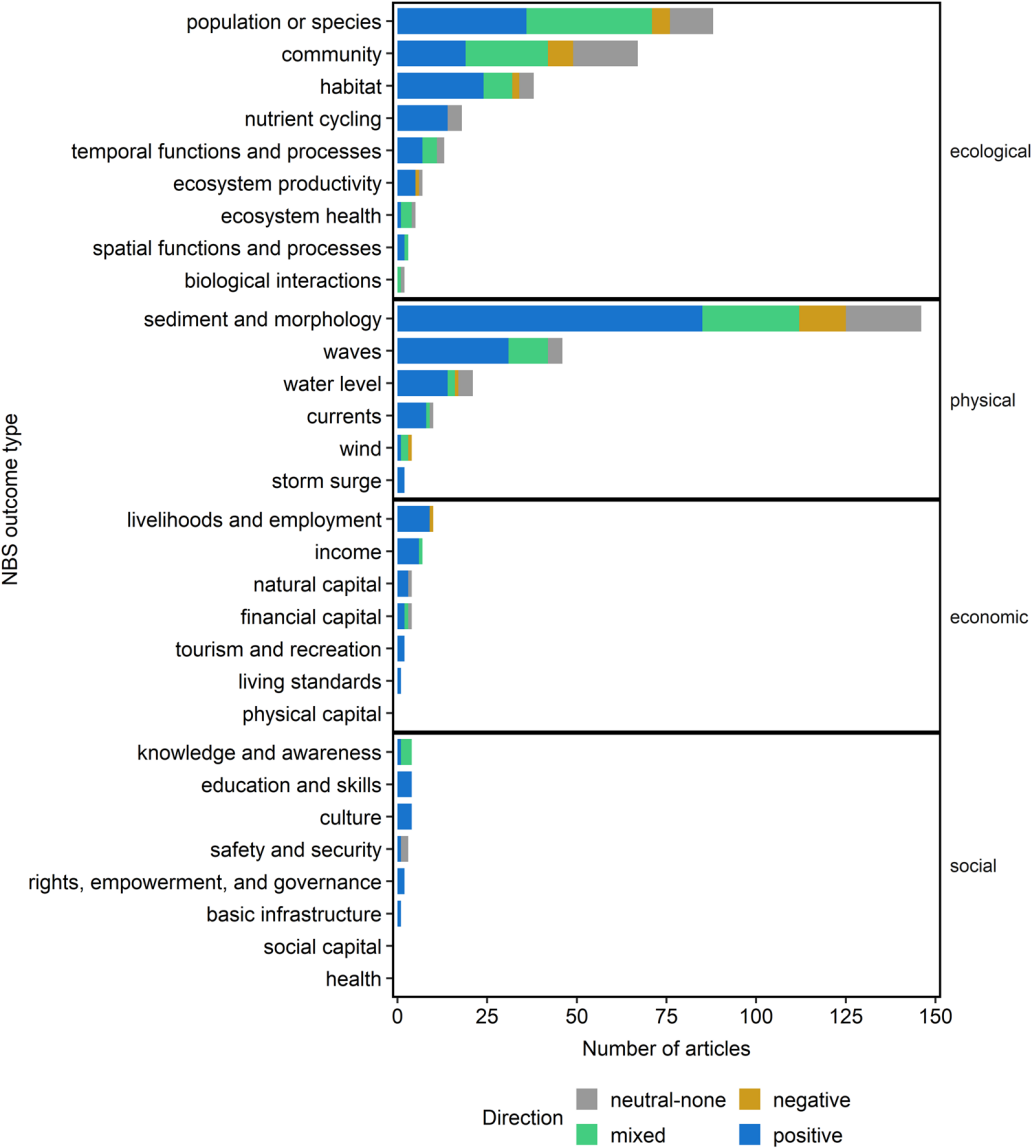
Number of articles by NBS performance outcome category. Outcome are faceted by whether the outcome is ecological, physical, economic, or social. Outcome types are colored by the outcome directionality (e.g., positive, negative); outcome directionality does not imply statistical significance. Some articles contained more than one intervention or outcome so can appear in more than one category within or across facets

More articles examined physical outcomes (50%; n = 202) and ecological outcomes (41%; n = 164) than economic (5%; n = 22) and social (4%; n = 14) outcomes (Fig. 6). The majority of evaluated ecological outcomes were related to population and species (n = 78), community (n = 64), and habitat (n = 37; see Table 5 for example metrics). Other ecological outcomes evaluated less frequently included those related to nutrient cycling (n = 18), temporal functions and processes (n = 12), ecosystem productivity (n = 6), and ecosystem health (n = 5). Two ecological outcomes, spatial functions and processes (n = 2) and biological interactions (n = 2), were rarely evaluated. The most common physical outcomes were sediment and morphology (n = 134) and waves (n = 45). Other evaluated physical outcomes included water level (n = 21) and currents (n = 10). Wind (n = 4) and storm surge (n = 2) outcomes were rarely evaluated. Compared to ecological and physical outcomes, sparser evidence existed on economic outcomes. Few articles evaluated economic outcomes: livelihoods and employment (n = 10), income (n = 7), natural capital (n = 4), financial capital (n = 4), and tourism and recreation (n = 2). Living standards was evaluated once, and physical capital was not evaluated. Social outcomes were the least evaluated outcomes compared to ecological, physical, and economic counterparts. Social outcomes included culture (n = 4), education and skills (n = 4), knowledge and awareness (n = 4), safety and security (n = 3). Rights, empowerment, and governance (n = 2), as well as basic infrastructure (n = 1), were rarely assessed. There was no evidence related to health or social capital.

**Table 5:** Common metrics used to assess NBS performance for A) ecological, B) physical, C) economic, and D) social outcomes

Category	Subcategory	Metrics
A) Ecological	Population or species	Abundance
		Biomass
		Catch per unit effort
		Density
		Size
		Mortality
		Growth
		Recruitment
		Survival
		Presence/absence
		Biological traits
		Cover (individual or population)
	Community	Community composition
		Abundance
		Diversity
		Evenness
		Richness
		Cover (community)
	Habitat	Cover
		Area
		Presence/absence
		Vertical relief
	Biological interactions	Species interactions
	Spatial functions and processes	Spatial distribution
	Temporal functions and processes	Colonization
		Succession
		Recruitment
		Resilience
		Growth over time
		Morality over time
	Ecosystem productivity	Primary production
		Secondary production
		Habitat function
	Ecosystem health	Toxin and contaminant distribution
	Nutrient cycling	Nitrogen concentration
		Carbon concentration
		Soil nutrient concentrations
		Denitrification
		Carbon sequestration
		Water retention
B) Physical	Waves	Wave attenuation
		Wave speed
		Wave energy
		Wave height
	Currents	Current speed
		Current magnitude
		Current dissipation
		Turbulence
	Winds	Wind speed
	Water level	Flooding level
		Flood risk
		Water surface elevation
		Tidal inundation
	Storm surge	Storm surge magnitude
	Sediment and morphology	Accretion
		Erosion
		Elevation
		Slope
		Depth
		Bulk density
		Sediment particle size
		Sediment composition
		Shoreline or habitat position
		Sediment transport or flux
		Sedimentation rate
		Sediment deposition
C) Economic	Income	Additional income
		Household income
		Individual income
	Livelihoods and employment	Livelihood benefits
		Job generation
		Percent labor
		Yield/production
		Business ventures
	Financial capital	Costs
		Savings
	Natural capital	Costs from NBS and services
		Willingness to pay
	Living standards	Poverty rate
	Tourism and recreation	Tourism income
	Safety and security	Hazard concern
		Emergency response capacity
D) Social	Education and skills	Education opportunities
		Technical training
		Student education attendance
	Knowledge and awareness	Community activities
		Response rates of community
		Individual and community perception
	Culture	Historical preservation
		Recreation opportunities
	Recreation and tourism	Scenic opportunities
	Basic infrastructure	Public service benefits
	Rights, empowerment, and governance	Social empowerment
		Community and household resilience

Directionality of ecological, physical, economic, and social performance outcomes varied, encompassing positive, negative, neutral, and mixed outcome directions (Fig. 6). For population or species outcomes, for example, there was a high number of cases with positive and mixed outcomes, followed by neutral and negative. Most reported physical outcomes were positive (e.g., reduced erosion rate, increased wave attenuation), yet there were examples of negative, neutral, and mixed outcomes, especially for sediment and morphology. Most reported economic outcomes were positive (e.g., reduced property damage costs), although there were several cases of mixed or neutral outcomes. Most social outcomes that were reported had positive directionality (e.g., increased educational or recreational opportunities), although safety and security also had neutral outcomes, and knowledge and awareness also had mixed outcomes.

Performance evaluations of NBS were conducted using a variety of metrics (Table 5). Common ecological metrics used to assess species and populations included abundance, biomass, density, and size, and community metrics included community composition, diversity, and richness (Table 5a). Physical metrics for sediment and morphology encompassed accretion, erosion, elevation, and sediment composition, whereas metrics for waves included wave attenuation, speed, energy, and height (Table 5b). Economic metrics included individual and household income, job generation, and poverty rate (Table 5c). Social metrics were assessed using metrics ranging from education and recreation opportunities to social empowerment and public service benefits (Table 5d).

A diversity of methods were used to evaluate NBS performance (Table 6). Ecological outcomes were assessed using visual transects, quadrat surveys, visual assessments, field measurements, field collections, and remote sensing approaches (Table 6a). Physical outcomes were measured using wave and current meters, water level loggers, and multiple sediment and morphology methods, ranging from core collection and surface elevation tables to quadrat surveys and elevation remote sensing (Table 6b). Economic and social outcomes were assessed using surveys sent to individuals, households, or select groups of constituents.

**Table 6:** Common methods used to assess NBS performance for A) ecological and B) physical, outcomes

Category	Subcategory	Metrics
A) Ecological	Population or species	Quadrat surveys
		Visual transects
		Visual assessments
		Photograph surveys
		Field measurements
		Field collections (nets, traps, cores)
		Passive acoustic monitoring
		Active acoustic mapping
	Community	Visual transects
		Field collections (nets, traps, cores)
		Visual assessments
		Quadrat surveys
		Photograph surveys
		DNA extraction
	Habitat	Quadrat surveys
		Aerial surveys
		Visual transects
		Habitat mapping
	Biological interactions	Experimental observations
	Spatial functions and processes	Visual transects
		Photogrammetry
	Temporal functions and processes	Quadrat surveys
		Visual surveys
		transect surveys
		Field measurements
	Ecosystem productivity	Field collections
		Laboratory processing
	Ecosystem health	Field measurements
		Field collections
	Nutrient cycling	Field collections (cores, sediment samples)
		Biogeochemical analysis
		Nutrient flux measurements
B) Physical	Waves	Wave meters and gauges
		Hydrodynamic measurements
		Pressure transducers
	Currents	Current meters
		Gypsum dissolution blocks
	Winds	Wind meter
	Water level	Water level meters and loggers
	Storm surge	Storm surge observations
	Sediment and morphology	Transect surveys
		Field collection (soil samples, cores)
		Elevation remote sensing
		Topographic surveys
		Surface elevation tables
		Sediment traps
		Radionuclide techniques
		Quadrat surveys
		Deposition measurements (feldspar)
		Aerial surveys

Evaluations of NBS performance outcomes were more frequently conducted over short temporal durations than longer time series (Fig. 5d). For instance, the majority of evidence stemmed from outcome evaluations conducted 1–5 years (n = 143) following the NBS intervention or less than 1 year (n = 110) following the intervention. Fewer studies reported outcomes measured 5 to 10 years (n = 44) or greater than 10 years (n = 36) following NBS establishment.

#### Intersection of NBS interventions and outcomes

##### NBS interventions versus ecological outcomes

**Fig. 7:**
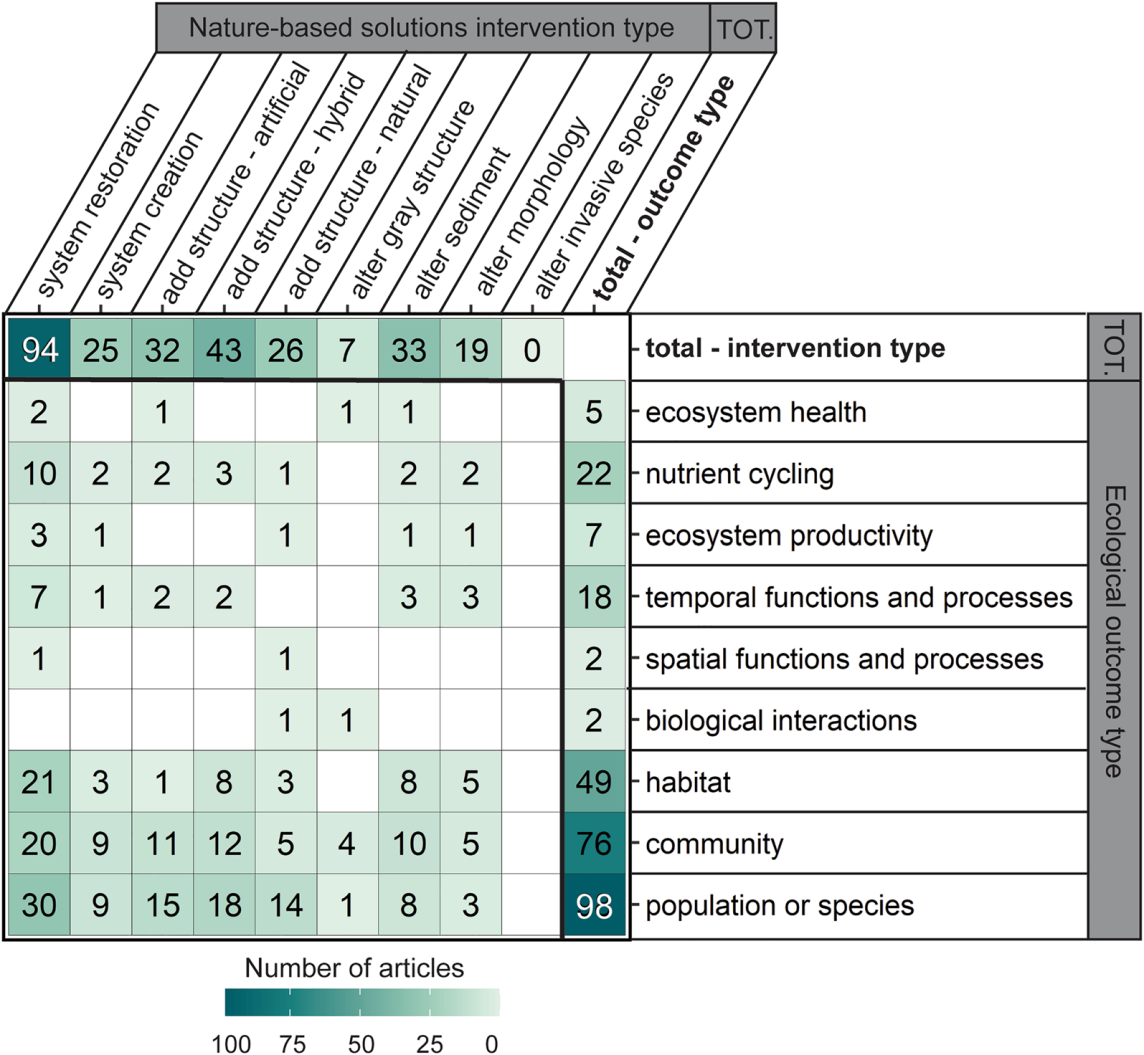
Distribution of evidence (number of articles) across NBS interventions and ecological outcomes. Some articles contained more than one intervention or outcome so can appear in more than one cell. Blank cells have zero articles. Total values across NBS intervention types and ecological outcomes are shown in the top row and far right column, respectively

Evidence clusters were most pronounced for ecological outcomes for NBS interventions that were restored ecosystems (n = 94), hybrid structure addition (n = 43), sediment alteration (n = 33), and artificial structure addition (n = 32; Fig. 7). For restored systems, the highest amount of evidence stemmed from population or species (n = 30) and habitat (n = 21), and community (n = 20) outcomes, whereas for added structures, evidence clusters occurred for population or species (n = 18 hybrid; n = 15 artificial) and community outcomes (n = 12 hybrid; n = 11 artificial). There was also a moderate amount of evidence for structure additions of natural origin for population or species outcomes (n = 14). There was some evidence on temporal functions and processes, as well as nutrient cycling, especially for restoration interventions. Across all NBS interventions, there was sparse to no evidence on spatial functions, biological interactions, ecosystem health, and ecosystem productivity.

##### NBS interventions versus physical outcomes

**Fig. 8:**
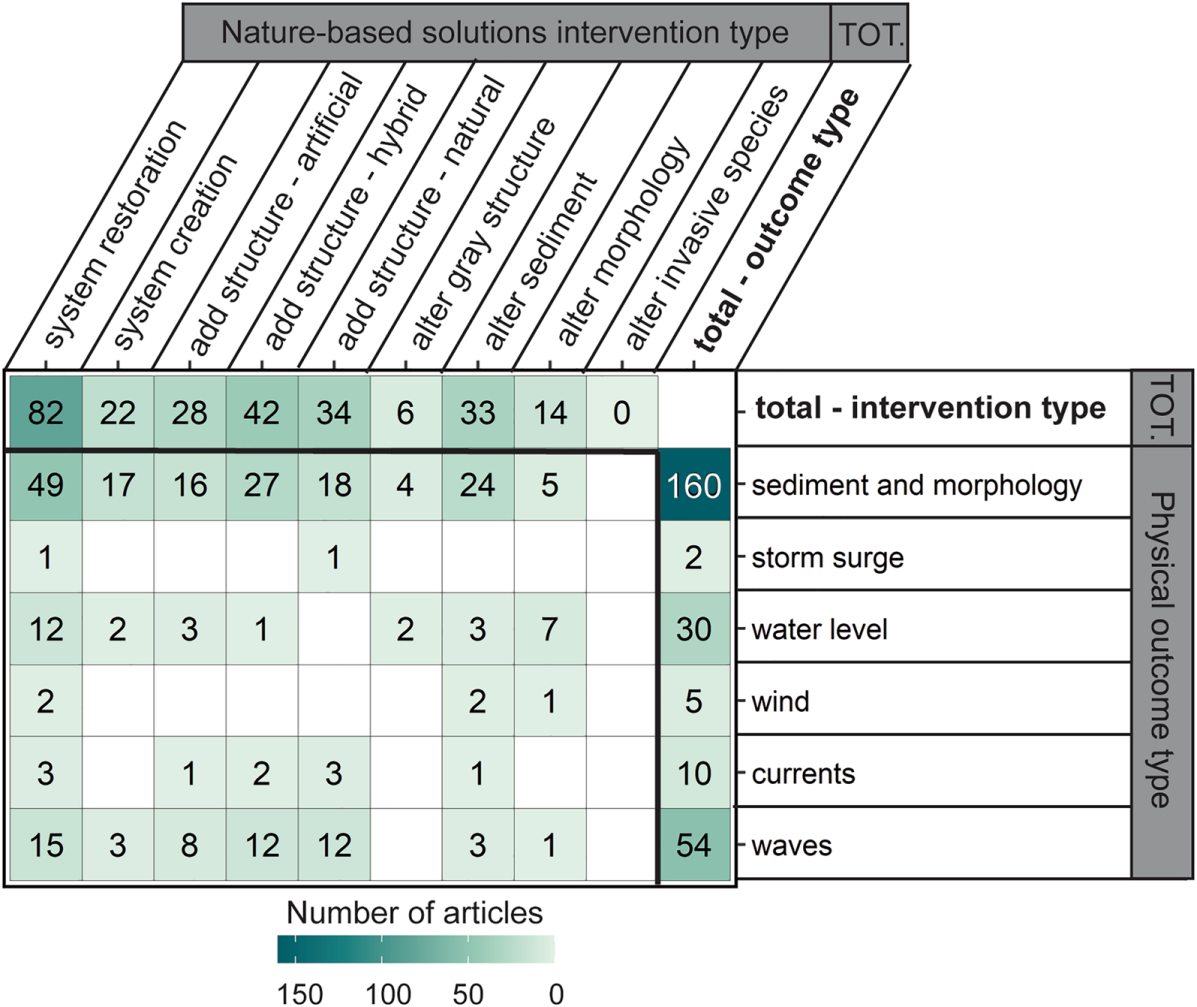
Distribution of evidence (number of articles) across NBS interventions and physical outcomes. Some articles contained more than one intervention or outcome so can appear in more than one cell. Blank cells have zero articles. Total values across NBS intervention types and physical outcomes are shown in the top row and far right column, respectively

Evidence on physical performance of NBS was highest for restoration (n = 82), hybrid structure addition (n = 42), natural structure addition (n = 34), and sediment alteration (n = 33), but most of these evaluations focused on sediment and morphology, water level, or waves (Fig. 8). For example, the evidence on physical performance for restored systems related to sediment and morphology (n = 49), waves (n = 15), and water level (n = 12), and evidence on hybrid structure additions focused on sediment and morphology (n = 27) and waves (n = 12). There was some evidence on currents for restoration (n = 3) and natural structure addition (n = 3). Sparse evidence existed for wind (n = 5) and storm surge (n = 2) across all NBS intervention types.

##### NBS interventions versus economic outcomes

**Fig. 9:**
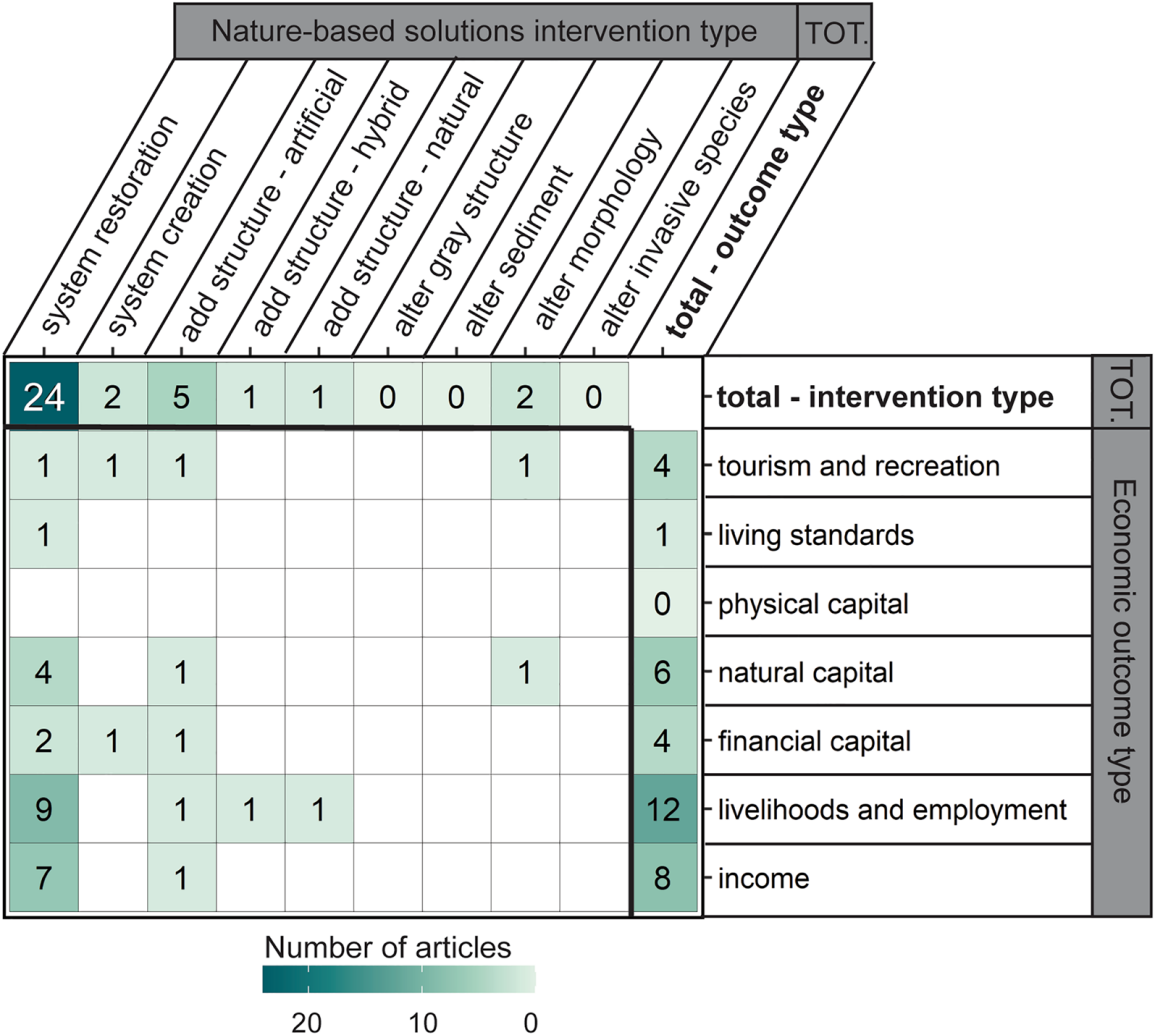
Distribution of evidence (number of articles) across NBS interventions and economic outcomes. Some articles contained more than one intervention or outcome so can appear in more than one cell. Blank cells have zero articles. Total values across NBS intervention types and economic outcomes are shown in the top row and far right column, respectively

Economic outcomes were assessed for restored systems (n = 24), artificial structure additions (n = 5), created systems (n = 2), morphology alterations (n = 2), hybrid structure additions (n = 1), and natural structure additions (n = 1; Fig. 9).Within restored NBS interventions, the evidence clustered on livelihoods and employment (n = 12) and income (n = 8). Across most NBS intervention types and economic outcomes, there were evidence gaps. For instance, there were no economic outcomes for alteration of gray structures, sediment alteration, or alteration of invasive species.

##### NBS interventions versus social outcomes

**Fig. 10:**
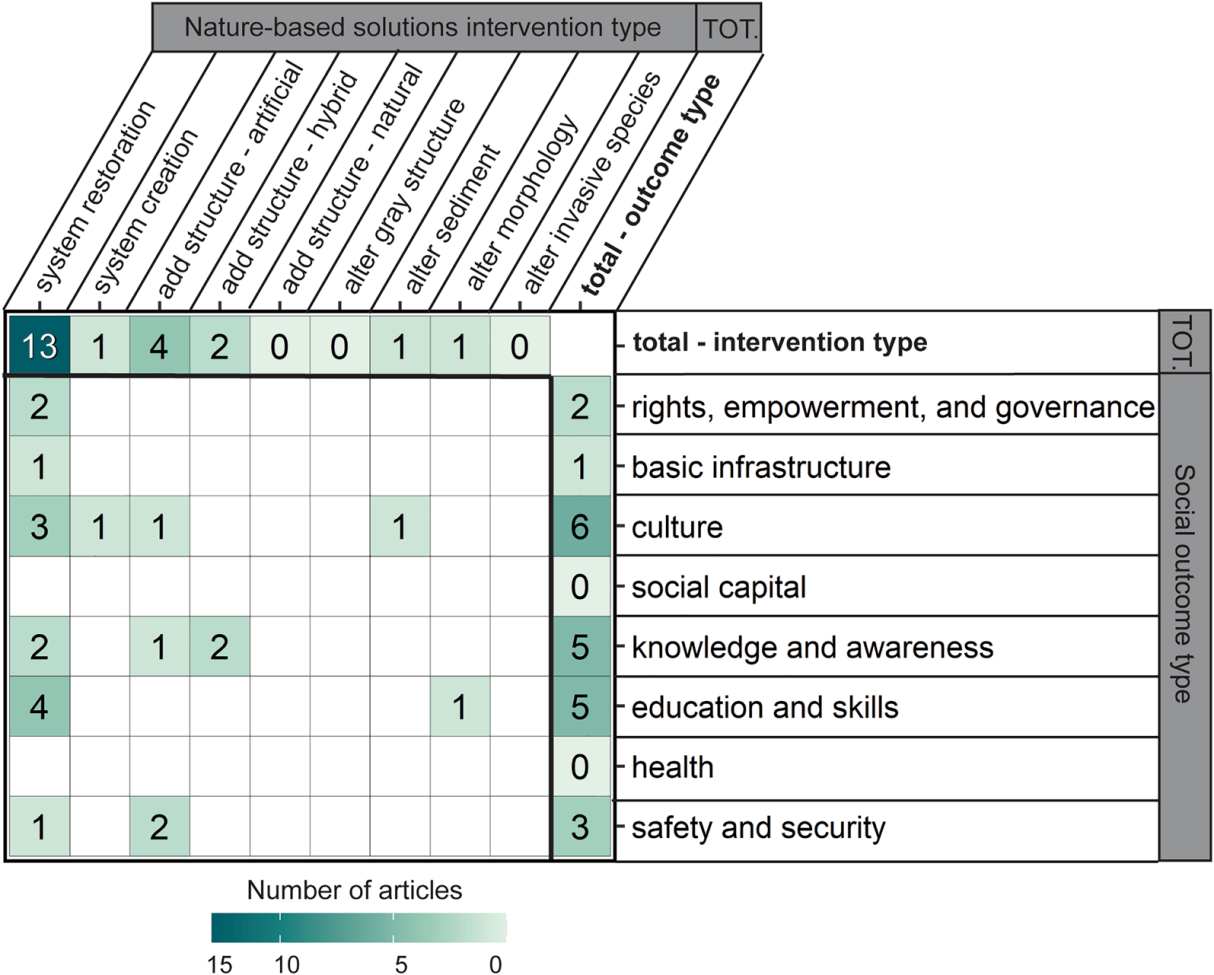
Distribution of evidence (number of articles) across NBS interventions and social outcomes. Some articles contained more than one intervention or outcome so can appear in more than one cell. Blank cells have zero articles. Total values across NBS intervention types and social outcomes are shown in the top row and far right column, respectively

Similar to economic outcomes, social outcomes were sparsely evaluated across NBS intervention types (Fig. 10). Restored systems (n = 13) exhibited moderate evidence related to education and skills (n = 4) and culture (n = 3), whereas added artificial structures had sparse evidence across multiple social outcomes. Other NBS interventions had zero to two social outcomes evaluated; no studies evaluated health or social capital.

## Discussion

### Evidence clusters and gaps

The systematic map details the published evidence base on the ecological, physical, economic, and social performance of NBS for coastal protection from 1980 through the early 2020s. The map reveals patterns in the distribution and abundance of evidence on NBS performance by publication traits (e.g., publication type, year, geography), ecosystem type, NBS intervention type, coastal protection context, study type characteristics (e.g., spatial scale, study design, cost reported), as well as a diverse suite of outcomes. In particular, the compiled systematic map highlights evidence clusters related to ecological outcomes (e.g., population and species, community, habitat), physical outcomes (e.g., sediment and morphology, waves), ecosystem types (salt marsh, mangrove), and particular types of NBS interventions (e.g., restoring and enhancing systems, adding hybrid and artificial structures). These evidence clusters lie in stark contrast to pervasive evidence gaps related to most economic and social performance outcomes, select ecological (e.g., ecosystem health, spatial functions and processes) and physical outcomes (e.g., storm surge, wind), some ecosystem types (e.g., seagrass, kelp), as well as for some types of NBS interventions, like altering invasive species or morphology. These findings on evidence clusters and gaps can help guide future research and management of NBS for coastal protection so that these interventions can be best designed, sited, constructed, monitored, and adaptively managed to maximize co-benefits. Here, we place our findings on types of NBS for coastal protection and associated performance outcomes into broader context, discuss implications, and highlight gaps and limitations of the systematic map.

#### Types of NBS for coastal protection

Our findings indicate that multiple types of NBS are used within coastal protection contexts. Actions intended to restore, enhance, or rehabilitate natural habitat or ecosystems and associated services were the most common types of NBS. Given the growing body of literature documenting the capacity of natural ecosystems to provide coastal protection benefits, it is logical that restoring or enhancing natural systems would be a popular approach towards achieving coastal protection goals. The finding that the majority of the evidence base on NBS interventions relates to restoration fits within the context of increasing marine restoration efforts and investments globally [3]. Adding artificial or hybrid structures to existing ecosystems represented another common NBS intervention. The popularity of these approaches coincides with increasing emphasis on how built structures can provide coastal protection benefits, as documented in field studies [76] and a recent evidence synthesis on coral and built structures [70]. Interventions to stabilize, remove, or place sediment were also common, which is not surprising given popularity of beneficial reuse of dredged sediment [15, 106] and managed realignment [50, 88] techniques. Several types of NBS interventions were less well studied and thus ripe for future research; these include alteration of morphology, alteration of gray infrastructure, and alteration of invasive species.

The NBS interventions within the evidence base included a mixture of those with stated goals of providing coastal protection, those that may not have identified an explicit goal but did measure the coastal protection capacity, and those that had both a stated goal and measured outcome. This suggests that while some NBS are designed and installed specifically to meet coastal protection goals, other NBS are evaluated for coastal protection capacity even though they may *not* be designed, sited, or installed to provide such services. This mismatch between intended goals and measured outcomes (e.g., 20% of studies did not have coastal protection as a goal in the associated publication but did evaluate coastal protection outcomes) highlights an opportunity to strategically design NBS for coastal protection from project inception, rather than to measure potential coastal protection co-benefits as an afterthought. This distinction is important, as there are often tradeoffs in restoration designs based on specific project goals. For example, a seagrass restoration design for coastal protection might plan to maximize the linear extent of the seagrass bed parallel to the shoreline, whereas seagrass restored primarily for biodiversity provisioning may try to optimize interior habitat space over linear extent, which could compromise the coastal protective services provided. Nevertheless, valuable lessons for coastal protection can be learned from NBS not originally intended for coastal protection, or those NBS that are not explicitly identified in the literature as intended for coastal protection. In these cases, rigorous evaluations across diverse suites of response variables are imperative, as well as the acknowledgement that the coastal protection capacity of NBS intended for vs. incidentally serving as coastal protection may differ [49]. Findings from our systematic map on coastal protection complement calls from previous review studies for interdisciplinary NBS design, implementation, and management of NBS to most effectively provide coastal protection benefits [60, 65, 72].

Given that this was a systematic map instead of a systematic review, we did not quantitatively assess effect sizes or performance of NBS interventions. We did, however, categorize the evidence base by outcome directionality—positive, negative, neutral, or mixed (both positive and negative). The evidence base included many more studies for which positive outcomes were reported than negative, neutral, or mixed. A possible explanation is that this may reflect a reporting or publication bias, where positive results are often more likely to be published than negative, mixed, or neutral findings [63, 64], adding further support to calls by Narayan et al. [65] for studies to report on negative outcomes associated with project failures or underperformance because of their value in understanding why NBS was not effective. Outcomes related to sediment and morphology, however, which are intimately tied to coastal protection, did report negative, mixed, and neutral findings. Similarly, results besides those that were positive were reported for factors like waves, water level, and currents. This suggests that in some cases, NBS may meet coastal protection goals but in others NBS may underperform.

#### Performance outcomes of NBS for coastal protection

A key result from our systematic map is that ecological and physical outcomes are often assessed as NBS for coastal protection. In contrast, social and economic metrics are not frequently evaluated and represent significant evidence gaps. This discrepancy has been previously highlighted for living shorelines [87], coastal nature-based defenses [65], and for NBS in terrestrial and marine environments for climate change [62, 99], as well as in restoration ecology [27]. We hypothesize that social and economic evidence related to NBS for coastal protection may reflect the funding landscape, where NBS installation and subsequent ecological and physical monitoring are emphasized over quantifying economic and social outcomes. Increasing calls for and funding opportunities to create equitable NBS provide a roadmap and “bright spots” for filling these gap [104]. We also posit that there may be a time lag to quantify social and economic outcomes associated with NBS that may take upwards of 5 to 10 years and may be incongruent with the demand for results in the short-term.

Among ecological performance outcomes, several outcomes were repeatedly assessed across studies (e.g., population and species, community, habitat), whereas outcomes related to the broader ecosystem health and functioning, as well as patterns and processes, were less well documented. This presents an opportunity to move beyond typical observational field measurements that document patterns and instead discern underpinning ecological mechanisms and processes. One approach for garnering this more holistic ecological understanding of NBS is to incorporate ecological theory into planning, assessment, and management of NBS, as has been called for in restoration science [84]. Similarly, there was an emphasis among physical outcomes on sediment and morphology (e.g., accretion, erosion), waves, and water level, but broader-scale outcomes like currents and winds were not well assessed. The outcomes were often assessed over short time scales of < 1 year to 5 years and local geographic scales, bolstering calls to develop standardized and holistic monitoring approaches for NBS [52], including those that harness advanced technologies [79]. Future research could invest in collecting longer term monitoring data on NBS performance and conducting regional studies that can help elucidate regional patterns and drivers.

### Limitations of the map

There are several potential sources of bias in our systematic map. First, our search was conducted for articles in the English language. Because of this language constraint, we were unable to conduct full text screening on 36 non-English articles. Our efforts to reduce bias included ensuring that our call for stakeholder contributed literature was shared with individuals outside of English-speaking countries, including Belgium, Brazil, Chile, China, Netherlands, South Africa, and Singapore. We also ensured that our search for literature in organizational websites included organizations that span English and non-English speaking countries, such as the Asian Development Bank, Duetsche Gesellschaft fur Internationale Zusammenarbeit, European Union, International Monetary Fund, International Union for Conservation of Nature, United Nations, and World Bank. Future evidence synthesis efforts could broaden the evidence base by incorporating non-English articles. We also limited eligibility to only certain types of ecosystems, and future syntheses could examine additional ecosystems.

Second, we conducted our title and abstract screening using Swift Active Screener, which uses a machine learning algorithm to incorporate screener feedback and predict articles that are relevant versus irrelevant. This software has been validated through multiple studies [44] and recently used for another systematic map [70]. To mitigate the potential bias from using machine learning to assist in title and abstract screening, we double screened the first 2,300 titles and abstracts in Swift Active Screener. Following double screening, we single screened titles and articles until the “recall rate” reached 95%. Despite these measures, it is possible that articles may have been overlooked by using machine learning to assist in this stage of the systematic map process.

Third, we conducted single screening for some portions of the systematic map process. After double screening 2300 titles and abstracts, we reverted to single screening. Additionally, we conducted single screening for full texts. To mitigate potential bias, we held rigorous screener training sessions, evaluated inter-reviewer consistency, and conducted quality assurance and quality control that included rescreening 5% of the full text articles and spot checks of all included articles.

## Conclusion

### Implications for policy and management

This systematic map highlights opportunities for policy makers and managers to develop initiatives for “end-to-end” management of NBS. This would include using data driven approaches to best design, site, construct, monitor, and adaptively manage NBS. Such a data driven approach could help ensure that NBS intended to meet coastal protection goals actually perform as intended. Other opportunities exist to leverage and encourage multidisciplinary collaboration to accelerate progress on improving NBS performance. Multidisciplinary approaches will be particularly valuable to garner a more comprehensive understanding of NBS performance not only ecologically and physically, but also economically and socially and to do so equitably across geographic regions.

More specifically, this systematic map provides a tool that policy makers and managers can use to prioritize and guide future initiatives related to NBS for coastal protection. The identified knowledge clusters and gaps can help inform prioritization of research directions and funding initiatives to, for example, fund initiatives that fill knowledge gaps or produce further systematic reviews and quantitative analyses on areas of evidence clusters. The map can also be used in development of standards and guidelines or “best practices” for NBS projects. For instance, the map suggests that best practices include monitoring a suite of ecological, physical, economic, and social data so that projects designed for coastal protection can also collect sufficient information on co-benefits or unintended consequences. Managers interested in implementing NBS may use the map to learn what worked, what challenges were faced, and how information was reported from similar NBS projects in their target geographic region, ecosystem type, NBS type, or another category. Managers overseeing current NBS or previously implemented NBS may also be able to use the systematic map to find examples of successful (or unsuccessful) NBS that can be used to help guide adaptive management or adjustment of their NBS strategy, if warranted.

### Implications for research

Our systematic map findings suggest several key opportunities for future research. Notably, there are large evidence gaps related to NBS performance: (1) for economic and social outcomes, (2) in kelp, coral reef, and seagrass ecosystems, and (3) for specific types of NBS like invasive species modification and alteration of gray infrastructure that future efforts can help fill. Future research studies should take care to monitor NBS performance over broad spatial (e.g., multiple projects over broad spatial scale – national, regional, global) and temporal scales, especially since much of the current evidence base stems from local and short-term studies, respectively. A particularly promising path for future research would be to form multidisciplinary teams that can not only examine ecological and physical outcomes of NBS but also social and economic outcomes. For example, teams could include ecologists and biologists, oceanographers, geologists, geographers, sociologists, economists, policy makers, and managers. Such highly collaborative endeavors could help ensure that NBS performance evaluations examine co-benefits and potential unintended consequences of NBS in both natural and human realms. Another future research direction is to use this systematic map for a systematic review. There is likely sufficient evidence on the ecological and physical performance of NBS in salt marsh, mangrove, and shellfish reef ecosystems to conduct quantitative syntheses. There may also be sufficient evidence to compare performance across the three types of NBS with added structure (artificial, hybrid, natural), as well as restoration interventions. There is likely not enough evidence to conduct a quantitative synthesis on economic or social outcomes.

## Supplementary Information

Additional file 1. ROSES reporting form

Additional file 2. Search strings

Additional file 3. Benchmarking articles

Additional file 4. Organizational website searches

Additional file 5. Typologies for interventions

Additional file 6. Typologies for outcomes

Additional file 7. Codebook and data extraction spreadsheet

Additional file 8. Included articles

Additional file 9. Excluded articles and exclusion reasons

Additional file 10. Coded data for included studies

## Data Availability

No datasets were generated or analysed during the current study.
